# Why do experts miss AI’s errors? Evidence from a randomized labeling experiment

**DOI:** 10.1093/pnasnexus/pgag146

**Published:** 2026-06-09

**Authors:** Sofoklis Goulas, Rigissa Megalokonomou, Panagiotis Sotirakopoulos

**Affiliations:** Research Team, foundry10, 4244 University Way NE, #85538, Seattle, WA 98145, USA; MacMillan Center, Yale University, 115 Prospect Street, New Haven, CT 06511, USA; Department of Economics, University of Crete, Gallos Campus, Rethymno 74 150, Greece; IZA@LISER — Institute of Labor Economics, Schaumburg-Lippe-Strasse 5–9, Bonn 53113, Germany; IZA@LISER — Institute of Labor Economics, Schaumburg-Lippe-Strasse 5–9, Bonn 53113, Germany; Department of Economics, Monash University, 27 Sir John Monash Drive, Caulfield East, Melbourne, VIC 3162, Australia; School of Economics, University of Queensland, St Lucia, Brisbane, QLD 4072, Australia; CESifo, Poschingerstrasse 5, Munich 81679, Germany; Faculty of Business and Law, Curtin University, Kent Street, Bentley, WA 6102, Australia; ARC Centre of Excellence for the Elimination of Violence Against Women, Curtin University, Kent Street, Bentley, WA 6102, Australia

**Keywords:** AI in education, human–AI collaboration, oversight of algorithmic systems, randomized labeling experiment

## Abstract

As organizations increasingly rely on algorithmic decision aids, human oversight is vital to prevent automated errors from spreading. But what makes experts correct an algorithm or let it stand? In a preregistered randomized experiment, education experts reviewed identical student work paired with an intentionally inaccurate score labeled as either human- or AI-generated. We independently varied whether the score was too harsh or too lenient. The outcome—the grading fairness gap—measures the distance between the expert’s revised mark and the objective rating. Under a harsh recommendation, the gap was 22% larger when the score was labeled as AI-generated; in the lenient case, the fairness gap under AI and human labels was statistically indistinguishable. Mediation analysis reveals that higher perceived ability and responsibility of the algorithm in the harsh scenario explain over half of the effect, while weaker attributions in the lenient case lead to stricter corrections. Thus, deference to AI depends not on automation itself but on the direction of its errors and the credibility it signals—offering design insights for accountable human–AI collaboration.

Significance Statement From legal advice to medical triage, AI assistants promise faster and more consistent decisions—provided humans still catch their errors. We tested whether experts can detect AI-generated grading errors—an everyday but high-stakes task. In a preregistered experiment, teachers audited obviously incorrect scores initially assigned by either a human colleague or an algorithm. Teachers corrected lenient mistakes equally well but were significantly less likely to adjust unduly harsh marks when the grade was labeled as AI-generated. Deference was strongest among younger, highly educated, and tech-confident teachers—precisely the group often viewed as early adopters of AI. The results reveal a blind spot in human oversight and highlight the need for calibrated training and transparent algorithmic design to ensure that decision-support systems improve, rather than distort, high-stakes judgments.

## Introduction

The rapid advancement of AI has sparked transformative shifts across sectors ranging from healthcare and finance to transportation and public administration (1–4). As AI systems take on increasingly complex decision-making tasks, their successful integration depends not only on technical performance but also on how effectively they complement and extend human capabilities (5). Especially for tasks that require expertise, consistency, or objectivity, individuals may rely on algorithmic guidance and trust AI recommendations over human ones (6–11).

In high-stakes domains, such as medical diagnostics or judicial recommendations (12, 13), the role of human oversight is indispensable: humans must be able to interpret, question, and, when appropriate, override algorithmic outputs (14). Human oversight of AI systems is also critical for mitigating algorithm aversion (15, 16). Ensuring that such oversight mechanisms are robust is a prerequisite for realizing the benefits of AI without compromising accuracy, accountability, or public trust (17). Education provides a tractable and policy-relevant setting for studying AI oversight (18–20). Grading decisions involve evaluative judgment, have real consequences for individuals, and enable clean experiments of whether humans accept, revise, or override algorithmic advice.

AI promises faster and more consistent grading, yet it also raises questions about human oversight. Widely used engines—C-rater, E-rater, Intelligent Essay Assessor, IntelliMetric, and LightSIDE—can misscore or embed bias, so teachers must still spot and correct errors. In classrooms, educators are both the primary users of these tools and the decision-makers who shape student outcomes (21). Their capacity to scrutinize algorithmic marks is vital to safe human–AI collaboration (22), but we know little about whether teachers can perform this oversight role. Existing studies of teachers’ attitudes toward student use of AI say little about educators’ own reactions to algorithmic support tools (23). This study fills that gap by offering the first experimental evidence on whether teachers can spot and correct inaccurate grades labeled as generated by AI systems. In the high-stakes setting of teacher grading, we compare educators’ responses to AI- vs. human-labeled scores and probe the psychological mechanisms that mediate those responses. The findings shed new light on how human oversight operates in practice and identify the conditions under which it is most likely to succeed. Because grades shape students’ educational trajectories, erroneous grading can distort human capital accumulation and contribute to labor market misallocation and inequality.

We examine how algorithmic grading recommendations shape expert teachers’ marks through a survey experiment. Participants view scenarios of identical student responses to open-ended questions, each paired with a plausibly incorrect score that is either inflated or deflated relative to an objective benchmark. The score’s stated labeled source—AI system or human colleague—is randomly assigned (24). Crucially, we also randomize the direction of the error, something prior algorithm-aversion studies did not do (25). The direction of error—harsh vs. lenient—is created simply by changing the number of correct answers in the student script while keeping the recommended mark fixed at 5/10. When the work actually merits, say, 8 points, the 5/10 score is harsh (under-grading); when it merits 2 points, the same 5/10 score is lenient (over-grading). After reviewing the work, teachers assign their own score. Comparing the deviation between each teacher’s score and the objective benchmark reveals whether the source of the initial grade affects their ability to detect and correct errors. The design thus tests whether teachers place greater weight on human or algorithmic advice when judging student work.

Our main result is that algorithmic recommendations significantly increase the grading fairness gap—a measure of how much a teacher’s assigned grade deviates from an objective benchmark—when they are harsh. This suggests that teachers are more likely to accept stricter grades from an AI than from a human. In contrast, when recommendations are lenient, algorithmic suggestions have no statistically significant effect on grading behavior, indicating that teachers place less weight on lenient AI inputs than on lenient human ones.

Human oversight of AI can falter because of cognitive biases, trust dynamics, and blurred role boundaries, all of which shape how users judge a system’s ability, intent, reasoning, fairness, and responsibility (26). Confidence in an algorithm’s competence is the strongest predictor of adoption. When educators believe a system produces accurate grades, they are more likely to follow its advice (27–29). Perceived intent also matters; users tend to comply when they think the system serves individual interests rather than organizational goals (30). Comprehension or rationale, which is made possible through transparency, explainability, and user control, enables informed oversight by clarifying the rationale behind an AI’s outputs (31, 32). Perceptions of fairness determine whether algorithmic judgments seem legitimate. This is especially the case in education, where context is critical. Finally, clear attribution of responsibility empowers users to override or challenge recommendations that clash with their professional values. Taken together, these dimensions show that effective human–AI collaboration depends not only on technical performance but also on how users interpret a system’s purpose and trustworthiness.

These mechanisms are particularly salient in education, where teachers increasingly oversee AI-generated outputs such as automated grades or feedback. When teachers trust the ability of the AI system they may be less inclined to scrutinize its output, even when it diverges from their own judgment. A lack of transparency can undermine comprehension and render teachers unable to justify or identify why a particular grade was assigned. Perceived unfairness, such as neglecting individual effort or contextual factors, can prompt rejection, particularly when fairness is central to teachers’ grading philosophy. Intent also matters: Teachers may resist recommendations they perceive as advancing bureaucratic or efficiency goals rather than student learning. And when responsibility for the final grade is ambiguous, professional agency may erode and lead to either passive acceptance or total disengagement. Even with discretion, teachers may be drawn to recommendations that validate their initial impressions, and thus reinforce preexisting biases rather than correcting them. Oversight in educational settings, then, depends not only on system accuracy but also on how educators interpret AI’s rationale and role within their professional responsibilities.

We test whether teachers’ perceptions of an AI grader’s intent, ability, comprehension, responsibility, and fairness explain its effect on the grading fairness gap. Perceived ability emerges as the pivotal mediator in both scenarios. When the algorithm proposes an unduly harsh 5/10, higher ratings of its ability—and, to a lesser extent, its responsibility—motivate teachers to leave the low mark largely intact, which widens the gap. When the algorithm proposes an inflated 5/10, skepticism dominates: Negative perceptions across all five dimensions prompt teachers to correct the score, and fully close the gap. Thus the credibility of algorithmic grading depends heavily on the recommendation’s direction. Teachers therefore accept a strict AI grade when the system is viewed as competent and accountable, even if fairness concerns linger. Lenient AI grades face a much higher bar: Unless the algorithm, simultaneously, scores well on intent, ability, fairness, comprehension, and responsibility, teachers override its advice and return to the fair grade.

Our study advances the literature on algorithmic accountability in three important ways. First, although previous research has called for human oversight to close accountability gaps in AI decision-making (33), there is little evidence regarding whether professionals can provide that oversight in practice (34). We offer an experimental test of this question in a student assessment and demonstrate how domain experts—ie teachers—respond to faulty AI recommendations. Second, by focusing on teachers who make consequential evaluative decisions, we pinpoint the limits of human oversight in educational AI and contribute concrete evidence to broader debates on structuring human–AI collaboration when accuracy and accountability matter. Third, we identify the mechanisms that drive effective oversight. Teachers’ corrections hinge on how they rate the algorithm’s ability and responsibility: They tend to accept harsh grades from an AI they view as competent and accountable, but reject lenient grades unless the same qualities are perceived. These asymmetries illustrate how perceived credibility can either reinforce or undermine human judgment.

This study has direct policy implications. First, the design of AI systems should emphasize perceived competence, fairness, and responsibility in order to build trust and promote informed oversight. Transparency and explainability are especially important when AI recommendations suggest leniency, because these are more likely to be scrutinized. Second, professional development could help train educators to interpret confidence signals, assess the rationale behind recommendations, and distinguish between appropriate adjustment and bias-driven override (35). Our results indicate that oversight depends on how recommendations are framed and how comfortable decision-makers are in engaging with them. Institutions seeking meaningful human oversight could therefore invest both in system features that facilitate interpretability and in structured training that builds evaluative fluency (36).

## Current study

The randomized experiment targeted in-service teachers across Greece. The sampling plan was prespecified in collaboration with local school authorities and preregistered in the AEA RCT Registry (AEARCTR–0015004). Invitations were distributed through official school e-mail lists, regional school boards, and professional fora to ensure broad coverage by school level, geographic region, and urban–rural status, as illustrated in Fig. S1. A €0.50 charitable donation per completed survey served as an incentive. All respondents taught in regular day schools. Evening, vocational, and special-education institutions were excluded.

A total of N=1,339 teachers were uniformly randomized into four vignettes defined by (i) the source of a deliberately erroneous score (human colleague vs. AI grader) and (ii) the direction of the error (harsh vs. lenient). Each teacher who completed the survey was randomly exposed to one of the four vignette scenarios though computerized randomization in Qualtrics. In each vignette, teachers viewed a subject-matched student exercise accompanied by a bullet-point list that explicitly labeled every one of the five answer elements as correct or incorrect, which enabled respondents to deduce the “fair” score before deciding whether to revise the preassigned grade of 5/10. The scenarios were as follows:

- **Harsh scenario.** Four answers were labeled correct and one incorrect (ie objective score=8/10).
- **Lenient scenario.** One answer was labeled correct and four incorrect (ie objective score=2/10).

**Figure 1:**
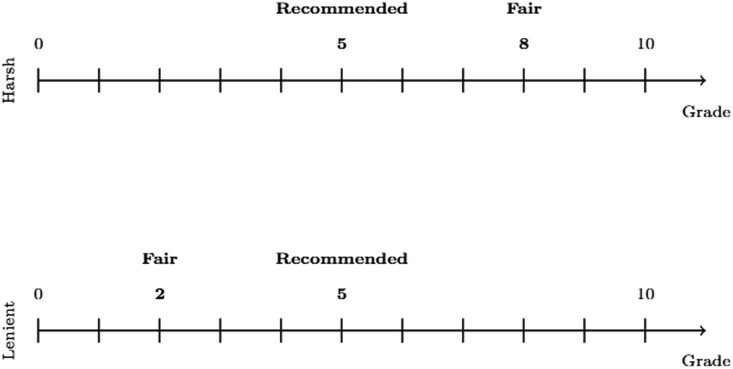
Fair and recommended grade across scenarios. This figure shows the recommended and fair grades (on a 0–10 scale) for both the harsh and lenient scenarios.

Thus, the recommended mark of 5/10 understated the true grade by three points in the harsh scenario and overstated it by three points in the lenient scenario (Fig. 1).

Our design builds on recent work using experiments in which information shown to participants is labeled as either human-made or AI-generated (24, 37, 38). To ensure realism, we convened a focus group of subject-matter experts—including three educators, one psychologist, and one communication specialist—to construct grading scenarios that reflect authentic classroom practice. The resulting vignettes capture plausible human-made grading errors. To parallel these, we also examined the types of grading mistakes produced by current LLMs (via targeted prompts to ChatGPT-4o) and verified that the AI-error vignettes closely mirror the error patterns commonly generated by modern AI graders. Our specific prompt was: “Can you grade the following exercise [copy paste the full text of the exercise].” Within each subject, all participants saw the same exercise, but we manipulated the label they saw (generated by a colleague of theirs or by an algorithmic grading system).

After reading the vignette, teachers entered the grade they believed the exercise deserved on a 0–10 scale, from which the primary outcome was computed. The primary outcome is the grading fairness gap, which measures the extent to which a teacher’s assigned grade deviates from an objectively determined “fair” grade. It is calculated as the absolute difference between the teacher’s grade and the fair grade when reassessing a sample exercise under treatment conditions:


$${\text{Grading fairness gap}}_{i}=\left|{\text{Teacher grade}}_{i}-{\text{Fair grade}}_{i}\right|$$

Taking the absolute value allows the measure to reflect the magnitude of the deviation regardless of whether the teacher graded too leniently or too harshly. The grading fairness gap is calculated once per teacher based on a single student exercise, which provides a clean, teacher-level measure of grading precision. By comparing each grade with the objective benchmark, the metric captures overall accuracy and reveals any bias introduced by the treatment (human vs. algorithmic recommendations). Although the sign of the gap shows the direction of bias, our main analysis relies on its absolute value to focus squarely on accuracy.

Teachers then completed five mediator items to gauge their perceptions of the grader’s *intent*, *ability*, *comprehension* of the rationale, *responsibility*, and *fairness* on a −5 (“completely disagree”) to +5 (“completely agree”) scale. The first two dimensions, *intent* (benevolence) and *ability* (competence), map directly onto core components of interpersonal and algorithmic trust (27–30, 39, 40). *Comprehension* is included because transparent, well-justified recommendations are more likely to be adopted (41–43) and to be judged as the product of a rational agent rather than an opaque “black box” (8, 44). *Responsibility* and *fairness* capture moral evaluations that often override performance considerations (45–47). Full item wording is provided in Appendix S2.

## Results

### Descriptive statistics

Table 1 presents summary statistics of participant characteristics across the human and algorithmic grader treatments, which indicate that the groups are well balanced and appropriate for comparison.^a^ We also conduct balance tests across the harsh and lenient treatment scenarios and report the results in Appendix Table S1. These tests reveal no statistically significant differences between groups, which reinforces the integrity of the randomization procedure.

**Table 1:** Respondent characteristics and balance tests.

	Human			Algorithm				
	Mean	SD	*N*	Mean	SD	*N*	Diff.	*P* value
Age (years)	49.7	9.5	573	49.8	9.5	596	0.077	0.889
Experience (years)	19.8	11.1	661	19.9	10.6	678	0.100	0.866
Years since graduation	27.1	10.1	661	26.9	10.1	678	− 0.228	0.679
Baseline leniency (0–10)	6.7	2.2	655	6.5	2.3	667	− 0.195	0.114

	%	*N*	%	*N*	Diff.	*P* value
Gender						
Female	68.8	661	70.1	678	1.224	0.627
Male	31.2	661	29.9	678	− 1.224	0.627
Education						
Bachelors	31.8	661	29.9	678	− 1.829	0.469
Masters	60.8	661	61.7	678	0.835	0.754
Doctorate	7.3	661	8.3	678	0.998	0.495
Studied abroad	23.3	572	22.9	594	− 0.356	0.885
Field						
Humanities	53.1	661	53.5	678	0.438	0.872
STEM	23.4	661	24.8	678	1.329	0.570
Primary/Other	23.4	661	21.7	678	− 1.768	0.440
Baseline Technological Literacy						

“I possess the necessary knowledge and appropriate tools to meet the modern technological demands of my role as an educator.”								
	Mean	SD	*N*	Mean	SD	*N*	Diff.	*P* value
Score	3.5	1.5	661	3.5	1.5	678	− 0.025	0.758

The table reports summary statistics for teachers across the treatment conditions *Human* and *Algorithm*. *Human* refers to the treatment condition in which a participant was exposed to a human recommender. *Algorithm* refers to a treatment condition in which a participant was exposed to an algorithmic recommender. Field denotes the teacher’s area of specialization and is categorized as follows: STEM, which encompasses subjects such as mathematics, physics, chemistry, science, and computer science; Humanities, which includes disciplines such as theology, art, sociology, and economics; and Primary/Other, which refers to educators involved in Greek language instruction, primary education, or teaching in technical institutions. *P* values stemming from a two-sample mean comparison *t* test are reported. Leniency is assessed on a scale from 0 to 10 based on the grades assigned by participants in the baseline exercises. Self-reported *Baseline Technological Literacy* is measured on a scale from −5 (Completely Disagree) to 5 (Completely Agree). SI Appendix, Fig. S4 plots the distribution of the *Baseline Technological Literacy* measure.

Appendix Fig. S2 presents the overall distribution of teacher-assigned grades. Appendix Fig. S3 builds on that view by plotting each grade against its corresponding gap, with marginal histograms that reveal where the largest deviations occur. Because the benchmark differs across scenarios, the scatterplots trace two mirror-image, piece-wise-linear patterns. In the harsh case, the benchmark sits near the top of the 0–10 scale; as a result, the data points form a downward-sloping diagonal that reaches its minimum (Gap=0) when teachers match that high benchmark and rises steadily as grades fall below it. Conversely, in the lenient case the benchmark is near the bottom of the scale, so the diagonal slopes upward: The gap is minimized when teachers reproduce the low recommendation and grows larger as they inflate the grade. The marginal histograms reinforce this visual impression.

Appendix Fig. S3 illustrates two key facts that motivate the subsequent analysis. First, teachers anchor strongly on the externally supplied recommendation, whether harsh or lenient, as evidenced by the dark clusters at the benchmark grade in both panels. Second, when teachers do deviate, they do so asymmetrically: Under the harsh benchmark they are inclined to soften grades (thus creating larger gaps for low marks), whereas under the lenient benchmark they tend to inflate grades (thus creating larger gaps for high marks). These behavioral asymmetries help explain why algorithmic recommendations can widen the grading fairness gap in one scenario but not necessarily in the other.

Appendix Tables S2–S6 show descriptive statistics for the mediators. In both harsh and lenient scenarios, teachers rate the source far less favorably when a recommendation is presented as “algorithmic” rather than “human,” with the largest gaps—around 2 points on the −5 to +5 scale—observed in the lenient condition for perceived ability, intent, fairness, and especially personal responsibility. Teachers revise these judgments when a *human* grader shifts from harsh to lenient, yet they do not adjust when the identical shift is attributed to an *algorithm*. This implies that algorithms are viewed as fixed rule-based entities whose qualities are insensitive to context.

### Impact of algorithmic recommendations on grading fairness

Table 2 examines the impact of algorithmic recommendations on the grading fairness gap, which is defined as the absolute deviation between a teacher’s grade and a benchmark (“fair” or “objective”) grade. The analysis is conducted separately for two scenarios: One in which the recommendation is harsh and one in which it is lenient. In the harsh scenario, the mean fairness gap is 1.386 when teachers are given human grading recommendations and 1.586 when they are given algorithmic grading recommendations. Regression estimates show that the algorithm increases the fairness gap by 0.302 points (P<0.01). In other words, exposure to an algorithmic grading recommendation increases the fairness gap by 22% relative to the 1.386-point gap under human advice. This suggests that teachers allow a harsh AI score to stand notably more often than the identical human score.

**Table 2:** Impact of algorithmic recommendation on grading fairness gap.

	Means		Without controls		With controls		
Scenario	Human	Algorithm	$\hat{\beta }$	*P* value	$\hat{\beta }$	*P* value	*N*
Harsh	1.384	1.584	0.264	0.022	0.300	0.003	677
			(0.039 to 0.490)		(0.104 to 0.496)		
Lenient	1.930	1.813	− 0.116	0.663	− 0.108	0.685	662
			(−0.639 to 0.406)		(−0.627 to 0.412)		

Parameter $\hat{\beta }$ is the estimated parameter of interest from a multiple regression specification. *Grading fairness gap* is the absolute difference between a teacher’s grade and an objective or benchmark grade, which is used to measure the extent of deviation from fair grading. A positive $\hat{\beta }$ indicates that the algorithmic recommendation increased the gap relative to the human recommendation; a negative $\hat{\beta }$ indicates it reduced the gap. All specifications include teacher field of specialization and county fixed effects. Specifications with controls include a comprehensive set of control variables, such as gender, age, level of education, teaching experience, years since graduation, an indicator for whether the participant studied abroad, baseline grading leniency, and indicators to reflect any missing values. Standard errors are clustered at county level. CI at the 95% level are reported in parentheses.

In contrast, in the lenient scenario, the algorithm leads to a slightly lower fairness gap on average (1.818 vs. 1.942 under human recommendations), but the difference is not statistically significant. The regression estimates are negative but small and not statistically significant ($\hat{\beta }=-0.120$, SE = 0.259). These results indicate that algorithmic recommendations significantly influence teacher grading behavior only when they are harsh. When algorithms recommend lenient grades, teachers discount them to the same extent as they do human-recommended lenient grades, as shown by a grading fairness gap statistically similar to that based on human recommendations.

To assess the robustness of our findings, we conduct a series of sensitivity analyses. Appendix Table S7 combines both scenarios in one model and includes an *Algorithm* × *Harsh* interaction. The estimated interaction coefficient is positive and statistically significant, and matches the separate-scenario results in Table 2. Second, we replace the absolute fairness gap with its signed counterpart, in which positive values indicate grades above the fair grade and negative values grades below it. This specification allows over- and under-grading to offset one another, and thus directly captures the direction of any bias. The estimated coefficients remain consistent in both magnitude and statistical significance with our baseline estimates (Table S8), which confirms that our results are not an artifact of taking absolute values. Next, we exclude out-of-range grades from our sample; specifically, those that exceed the fair grade in the harsh scenario or fall below it in the lenient scenario. This ensures that our estimates are not influenced by extreme or implausible grading behaviors. Again, the results are robust (Table S9). Finally, we explore alternative definitions of the fair grade by adjusting the benchmark by 1 point (ie fair grade ±1) in both scenarios. This robustness check accounts for potential subjectivity in determining what constitutes a truly “fair” or objective grade by allowing for a slightly broader interpretation of fairness. The results remain consistent in both magnitude and significance, which demonstrates that they are not dependent on a precise cutoff or definition of fairness. (Table S10).

### Differential effects of algorithmic recommendations on grading fairness

**Figure 2:**
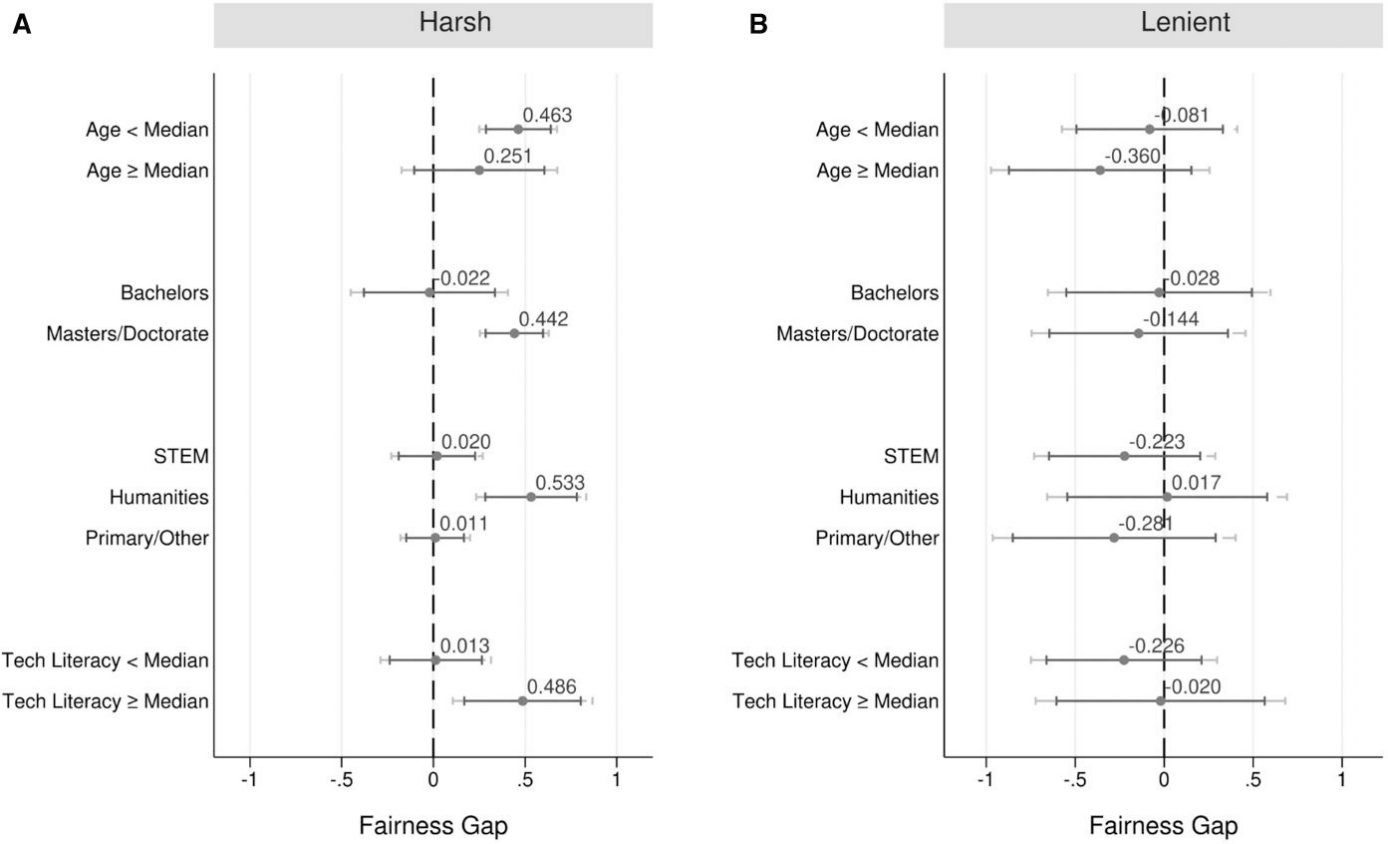
Differential results by teachers’ characteristics. This figure plots the estimated treatment effect (algorithm vs. human) on the grading fairness gap for teacher subgroups defined by age, education, field of specialization, and baseline technological literacy. Positive coefficients indicate that the algorithmic recommendation increased the gap relative to the human recommendation; negative coefficients indicate that it reduced the gap. A) The results for the harsh scenario and B) for the lenient scenario. Dashed and solid error bars represent 90% and 95% CI, respectively. Median age is 51. Median *Baseline Technological Literacy* score is 4. We present detailed estimates in Appendix Tables S18–S20.

We investigate whether the estimated effect varies across teacher subgroups (Fig. 2). When the AI understates the true score (harsh scenario), the algorithm–human difference is positive and statistically significant for teachers who are younger than 51 ($\hat{\beta }=0.463$, P<0.001), hold a master’s or doctoral degree ($\hat{\beta }=0.442$, P<0.001), specialize in the humanities ($\hat{\beta }=0.533$, P<0.001),^b^ or report high technological literacy ($\hat{\beta }=0.486$, P<0.05). By contrast, the gap is small and statistically indistinguishable from zero for older, bachelor-only, STEM, or low-tech-literacy teachers. Male and female instructors both exhibit a positive gap (men: $\hat{\beta }=0.394$; women: $\hat{\beta }=0.260$), but the gender difference itself is not significant (Fig. S8). Under the *lenient* scenario, in which the AI *overstates* the fair score, we detect no statistically significant algorithm–human differences in any subgroup. Likewise, the gap does not significantly vary by teaching experience, study-abroad background, school locale, or baseline grading leniency. Table S21 reports tests of heterogeneity in the impact of algorithmic recommendations across teacher subgroups using interaction terms. While the point estimates suggest heterogeneity across groups, the CI overlap for some comparisons (eg by age), indicating that the differences between subgroups should be interpreted cautiously.

### Why do algorithmic recommendations alter grading behavior?

We explore the mechanisms through which the recommendation source (algorithm vs. human) affects grading behavior. Specifically, we examine whether teachers’ perceptions of the evaluator’s ability, comprehension, fairness, intent, and responsibility help explain the effect of algorithmic exposure on the recommendation take-up.

Each mediator captures distinct yet overlapping facets of teachers’ decision-making. Appendix Table S11 reports Cronbach’s alpha coefficients for every mediator combination across the harsh and lenient scenarios. Reliability generally rises as additional mediators are combined: Most two-item pairs fall in the mid-0.60s to low 0.80s, three-item sets cluster in the mid-0.80s, and four-item groupings approach the high 0.80s. The greatest internal consistency appears when all five mediators—*ability*, *comprehension*, *fairness*, *intent*, and *responsibility*—are included (α=0.899 in the harsh scenario and α=0.907 in the lenient). Among pairs, *comprehension–intent* yields the lowest reliability (α≈0.66), whereas *fairness–responsibility* attains the highest (α≈0.90), which demonstrates substantial but not redundant overlap. No combination approaches the conventional redundancy threshold of 0.95, which suggests that these mediators capture complementary, rather than duplicative, dimensions that influence teachers’ grading decisions.

In both the harsh and lenient recommendation scenarios, teachers associate human evaluators with higher ability than algorithmic ones (see Figure S7 and Table S12). However, in the harsh scenario, this gap is smaller (Table S13). This pattern suggests that teachers perceive harsh algorithmic recommendations as less indicative of low ability than comparable human recommendations. In other words, harshness appears to function as a signal of evaluator competence (48).

Table 3 presents a mediation analysis of the grading fairness gap under both the harsh and lenient scenarios. Because the perception measures are collected after teachers observe the recommendation source, the results should be interpreted as suggestive evidence on the psychological channels through which the treatment operates rather than as fully causal mechanisms. This interpretation relies on the standard assumption that unobserved factors do not jointly influence teachers’ reported perceptions and their grading decisions.

**Table 3:** Mediation analysis.

	Mediators									
	Ability		Comprehension		Fairness		Intent		Responsibility	
Scenario	$\hat{\beta }$	*P* value	$\hat{\beta }$	*P* value	$\hat{\beta }$	*P* value	$\hat{\beta }$	*P* value	$\hat{\beta }$	*P* value
Harsh										
Indirect	0.218	0.000	− 0.008	0.328	0.027	0.199	0.057	0.149	0.143	0.016
	(0.106 to 0.329)		(−0.023 to 0.008)		(−0.014 to 0.069)		(−0.020 to 0.135)		(0.026 to 0.260)	
Direct	0.124	0.207	0.348	0.003	0.314	0.006	0.284	0.045	0.196	0.121
	(−0.068 to 0.315)		(0.118 to 0.579)		(0.092 to 0.535)		(0.006 to 0.562)		(−0.052 to 0.443)	
Mediator Mean	0.670		0.420		− 0.512		− 0.372		− 0.823	
Mediator SD	3.235		3.430		3.320		3.152		3.350	
Lenient										
Indirect	− 0.285	0.000	− 0.138	0.043	− 0.161	0.040	− 0.288	0.001	− 0.302	0.003
	(−0.394 to −0.175)		(−0.272 to −0.004)		(−0.315 to −0.007)		(−0.453 to −0.122)		(−0.498 to −0.106)	
Direct	0.112	0.633	− 0.035	0.840	− 0.021	0.907	0.114	0.625	0.120	0.525
	(−0.348 to 0.572)		(−0.376 to 0.305)		(−0.380 to 0.337)		(−0.342 to 0.570)		(−0.250 to 0.489)	
Mediator Mean	1.033		1.000		− 0.380		0.139		− 0.467	
Mediator SD	3.363		3.411		3.382		3.128		3.535	

Parameter $\hat{\beta }$ is the estimated parameter of interest from mediation analysis. A positive $\hat{\beta }$ indicates that the algorithmic recommendation increased the gap relative to the human recommendation; a negative $\hat{\beta }$ indicates that it reduced the gap. Mediator questions were presented after outcome questions in our survey instrument. *Grading fairness gap* is the absolute difference between a teacher’s grade and an objective or benchmark grade, and is used to measure the extent of deviation from fair grading. All specifications include a comprehensive set of control variables, such as gender, age, level of education, teaching experience, years since graduation, an indicator for whether the participant studied abroad, baseline grading leniency, and indicators to reflect any missing values. We also control for teachers’ field of specialization and include county fixed effects. Standard errors are clustered at county level. CI at the 95% level are reported in parentheses.

Under the harsh scenario, we find that ability and responsibility significantly mediate the effect of AI exposure on grading behavior. Specifically, the indirect path through *ability* is positive and significant ($\hat{\beta }=0.218$, P<0.01). This indicates that an AI label is associated with higher perceived grader competence relative to a human label, which in turn predicts a wider grading fairness gap. This single pathway accounts for roughly 73% (0.218/0.300) of the total AI-vs.-human effect, and thus renders perceived competence the largest identifiable driver of the harsher AI outcome. The indirect effect through *responsibility* is likewise positive and significant ($\hat{\beta }=0.143$, P=0.016), which indicates that when teachers view the AI grader as accountable for its decisions they allow harsher marks to stand, thereby widening the grading fairness gap. This pathway explains about 47% (0.143/0.300) of the total AI-vs.-human effect. In contrast, the indirect paths via *comprehension*, *fairness*, and *intent* are not statistically different from zero. At the same time, sizeable direct effects remain: Comprehension ($\hat{\beta }=0.348$, P=0.003), fairness ($\hat{\beta }=0.314$, P=0.006), and intent ($\hat{\beta }=0.284$, P=0.045) each retain a significant association with the fairness gap after all mediators are included. These residual direct effects suggest that part of the AI label’s influence may operate through additional, unmeasured channels beyond the five perceptions captured here. Appendix Tables S14 and S15 present the estimated parameter of interest under the harsh scenario when we sequentially include and exclude each mediator.

In the lenient scenario, each mediator—*ability*, *comprehension*, *fairness*, *intent*, and *responsibility*—significantly channels the effect of algorithmic exposure on teachers’ grading. Specifically, Table S15 shows that each mediator is positively associated with the grading fairness gap. Because teachers attribute lower levels of these qualities to algorithms than to human graders (Table S12), weaker perceived evaluator attributes for AI lead them to correct lenient algorithmic marks more aggressively, which yields fairer final grades. Formal mediation results confirm this mechanism: The indirect paths through *ability* ($\hat{\beta }=-0.285$, P<0.001), *comprehension* (−0.138, p=0.043), *fairness* (−0.161, p=0.040), *intent* (−0.288, P=0.001), and *responsibility* (−0.302, p=0.003) are all negative and significant. Thus, weaker attributions of evaluator qualities to the algorithm cause teachers to assign fairer final grades in the lenient scenario. Appendix Tables S16 and S17 present the estimated parameter of interest under the lenient scenario when we sequentially include and exclude each mediator.

Future experimental work could more directly test these pathways by varying observable signals of evaluator reliability—such as accuracy track records, accountability framing, or explanation quality—thereby isolating whether changes in perceived competence and responsibility causally alter teachers’ willingness to override recommendations.

### Teacher uptake and attitudes toward generative AI tools

At the end of the experiment, we administered a brief postsurvey module that asked teachers about their own use of generative AI tools (eg ChatGPT) and their views on algorithmic grading. Table S23 summarizes teachers’ self-reported behaviors and attitudes toward generative AI. Nearly half of respondents (48%) use AI tools at least weekly for lesson preparation, yet more than one-quarter (26%) have never tried them. When advising students, 41% recommend experimentation (18% “without reservations,” 24% “with reservations”). However, only 16% actively urge fellow teachers to adopt the technology; more than half (56%) rarely or never do so. Perceptions of algorithmic grading are tepid: On a −5 to +5 scale, the mean belief that AI can grade *fairly* is close to zero ($\bar{x}=0.03,\;\mathrm{SD}=2.81$). Average *willingness* to let AI grade is slightly negative ($\bar{x}=-1.03,\;\mathrm{SD}=3.18$), as is the perceived *ethical acceptability* ($\bar{x}=-1.33,\;\mathrm{SD}=3.03$).^c^ All in all, teachers are open to AI as a planning aid but remain skeptical about delegating evaluative authority to machines.

This skepticism is grounded not only in abstract concerns about fairness or control, but also in the nuanced realities of classroom life. At the end of the survey, teachers were invited to respond to an open-ended prompt: “Is there anything you would like to add or explain?” Some used this opportunity to elaborate on their views about the role of AI in grading. Their responses emphasized that grading often requires contextual sensitivity that AI systems lack. Teachers cited examples involving illness, learning difficulties, and students from vulnerable backgrounds—situations in which discretion and empathy are essential. As one asked, “Can AI perceive special circumstances, such as a student’s absence due to illness, a lack of teaching due to teacher absences, and therefore insufficient instruction?” Another stated, “Learning difficulties influence grading,” while a third explained that “teachers take into account parameters such as social conditions (eg how do I grade a child from Egypt who, if held back, will be married off at 12), and family environment (abused children, children of drug addicts, etc.).” Others pointed to subject-specific concerns, arguing that AI may be better suited for technical tasks than interpretive ones. One teacher remarked, “The artificial intelligence system lacks emotional flexibility; it is fair and accurate in science subjects. In humanities subjects, I think it just helps [as a tutor], it’s not [good] in grading.” Inductive coding of all comments that voiced reservations shows that 3 out of 4 highlighted moral or ethical blind spots: AI’s inability to attend to individual circumstances. Only 1 out of 4 focused on technical limits such as subject specificity. Because most concerns are ethical rather than mechanical, practices or policies that rely solely on greater algorithm accuracy are unlikely to overcome teachers’ objections (49).

## Conclusion

This study offers the first experimental evidence on experts’ ability to oversee and correct algorithmic input. Expert teachers were more likely to accept an unduly harsh grade when it was attributed to an AI compared to a human expert. This pattern suggests limited capacity for effective oversight: If experts miss visible grading errors, they are even less likely to detect subtler flaws in AI-generated instructional content. This concern extends beyond education. For example, similar oversight problems may arise when physicians review AI-assisted diagnoses or hiring managers evaluate algorithmic screening results in high-stakes contexts.

Our mediation analysis shows that the asymmetry between harsh and lenient AI grading scenarios is driven by perceptions of algorithmic competence and responsibility. Teachers respond more skeptically to lenient AI recommendations and their perceptions are fully explained by teachers’ lower ratings of the algorithm’s ability, comprehension, fairness, intent, and sense of responsibility. These findings underscore the fact that teacher reliance on AI is highly context-sensitive and shaped not only by the recommendation itself but also by how the algorithm is perceived in terms of credibility.

Our subgroup analysis shows that experts who are younger, more highly educated, or more technologically confident are especially prone to accepting an AI grader’s overly harsh assessment. Because these groups are often viewed as early adopters of new technology, this finding challenges the common belief that tech-savvy professionals automatically provide stronger human oversight. Humanities teachers—whose assessments often allow greater scope for subjective judgment—exhibit somewhat greater deference to AI’s harsh grades. This pattern is consistent with the idea that algorithmic advice may become more influential when evaluation criteria are more ambiguous. It also aligns with broader evidence that people may cede judgment to machines even on tasks they perceive as subjective (6). Our mediation analysis provides suggestive evidence that ability and responsibility perceptions are a stronger mediating channel among Humanities teachers (Table S22). Greater algorithmic deference in this group may reflect stronger responsiveness to judgment-relevant cues in domains where evaluation criteria are more subjective.

Our results have direct implications for education policy and the responsible use of AI in classrooms. First, algorithmic tools should be designed to signal both competence and accountability, so that teachers treat AI advice with the same critical scrutiny they apply to human guidance. Second, professional development programs may help prepare teachers to audit and, when necessary, override algorithmic recommendations. Training in bias detection, error patterns, and explainability can calibrate trust and improve the accuracy of human oversight. Treating educators as co-designers and critical partners—not passive end users—will help ensure that AI supports rather than supplants professional judgment and advances decision quality.

A limitation of the present study is that it may be restricted to the Greek context. At the same time, its insights may extend to other jurisdictions. Teachers and students worldwide now operate in “algorithm-saturated” environments: Social media feeds, adaptive learning platforms, and generative-AI assistants have normalized machine recommendations and raised expectations of their accuracy (18, 19). In settings in which technological familiarity is high but professional training in AI auditing is limited, the asymmetrical deference to harsh algorithmic judgments we uncover could reproduce, or even amplify, grading inequities. From an economic perspective, the costs of such grading errors are not merely distributive but efficiency-reducing: If some students are persistently undervalued, society risks underinvestment in their skills, inefficient sorting in the labor market, and the amplification of educational inequality.

The results should also be interpreted against the backdrop of rapidly changing educator engagement with generative AI. Survey evidence from our sample indicates that many Greek teachers already report using such tools in lesson preparation and hold heterogeneous but often cautious attitudes toward student and peer use (Table S23). Because generative AI adoption in education is recent and expanding, these responses likely reflect a transitional phase rather than stable long-term preferences. For this reason, the paper does not attempt to infer secular changes in attitudes. Instead, it isolates a more fundamental capability: whether educators can successfully monitor and correct algorithmic outputs when they encounter them. This distinction is important because even if acceptance of AI increases over time, the effectiveness of human oversight will remain a binding constraint on the safe deployment of automated grading systems.

The experimental task makes the correct grade relatively easy to infer using an explicit checklist, allowing the design to isolate label-driven reluctance to override an AI recommendation. The estimates therefore capture source-based deference rather than oversight under the informational uncertainty typical of real grading environments, where ambiguity, time pressure, and fatigue may either increase reliance on algorithmic advice or prompt stronger independent judgment. Real decision-support systems also provide explanations, confidence cues, accuracy histories, and institutional framing. Future work could test oversight behavior under richer informational and workflow conditions.

This study opens several avenues for future investigation. First, further research is needed to examine whether the greater deference to harsh than lenient AI recommendations we document extends to other evaluative tasks in education; these include formative assessment, student feedback, or performance monitoring. Second, future experiments can vary how much explanation the AI provides in order to learn whether clearer rationales prompt closer scrutiny. Future experiments could also vary the anchor grade and the magnitude of the grading error to assess whether the harshness asymmetry persists near pass–fail thresholds, under smaller deviations, or at more extreme recommendations. Finally, our experimental design can be applied in fields such as healthcare, justice, finance, and hiring to identify whether professionals show similar patterns of overreliance or skepticism when using AI. Together, these lines of inquiry can guide the responsible rollout of decision-support systems.

Our results underline the need for transparency, robust oversight, and context sensitivity in high-stakes settings such as grading, admissions, and hiring. Teachers accepted harsh AI grades more readily than comparable human grades, and thus risked unfair outcomes. Training, improved explainability, and close collaboration with end users may recalibrate trust and strengthen human–AI teamwork. Extending this approach to other expert domains will help ensure that AI augments rather than undercuts professional judgment. A user-first perspective that reflects the needs and limits of human overseers is essential for building AI tools that work reliably in real-world environments.

## Materials and methods

### Ethics statement

This study was reviewed and approved by Monash University’s Office of Research Ethics (Protocol #45983) and preregistered in the AEA RCT Registry (AEARCTR-0015004). The survey experiment was carried out online in collaboration with local school authorities and complied with the guidelines of the national Institute of Education Policy. All participating teachers received an information sheet that described the study and provided informed consent electronically before beginning the questionnaire.

### Questionnaire

The 21-question survey, which did not collect any personally identifiable information, is reproduced in Appendix S2. Native-speaking members of the research team translated the instrument from English into Greek to ensure both cultural and linguistic accuracy prior to distribution. The questionnaire consisted of four sections: (i) baseline teacher characteristics (including technological literacy), (ii) baseline leniency measures, (iii) randomized treatment blocks, and (iv) attitudes toward and use of AI tools. As part of the baseline measures, participants were asked to evaluate a sample exercise related to their field of expertise (mathematics and physics, science, or non-STEM) by assigning it a grade on a 0 to 10 scale. This grade served as a measure of each teacher’s baseline grading leniency. For subgroup analysis, teachers were categorized as “more lenient” or “less lenient” depending on whether their assigned grade was above or below the median score within their subject group. Questions related to teacher characteristics and key grading practices, such as technological literacy and grading leniency, preceded treatment exposure to ensure that responses were not influenced by participants’ reactions to the algorithmic or human grader. The teacher characteristics section collected information on age, gender, years of teaching experience, education level, time since graduation, study-abroad background, and field of specialization. To avoid overburdening participants early in the survey, more general questions about AI, such as current use, willingness to recommend such tools, and perceptions of fairness and ethicality, were asked after the treatment section. The median survey duration was 7.2 min.

### Sample representativeness

To assess external validity, we benchmark our survey sample against (i) the national population of K–12 teachers and (ii) the broader working-age population in Greece, using data from the Greek Census reported by the Hellenic Statistical Authority (ELSTAT). Appendix Table S24 summarizes the comparisons.

In terms of gender composition, women account for 69% of our respondents, a figure that closely mirrors the national distribution among K–12 teachers (72%), though it remains substantially higher than the 38% observed in the overall workforce. This alignment suggests that, along this dimension, the sample accurately reflects the structure of the teaching profession in Greece. At the same time, respondents are somewhat older, with an average age of 49 compared to 40 among K–12 teachers and 41 in the broader employed population. While this indicates a tilt toward a more senior cohort, the difference in average professional experience is modest. Survey respondents report 20 years of experience, compared to 18 years among K–12 teachers and 19 years in the workforce overall. This pattern is consistent with our focus on “expert” educators and suggests that the sample captures teachers with substantial institutional knowledge and classroom practice. Also, our survey sample is somewhat more highly educated than the benchmark groups. Eight percent of respondents hold a PhD, compared to roughly 2 percent of K–12 teachers and 0.6 percent of the overall workforce. This higher level of formal qualification further reflects a slightly higher concentration of experienced and academically credentialed educators in our survey.

Overall, the sample closely reflects the national teaching workforce in terms of gender and experience, while somewhat overrepresenting older and more highly educated educators. The findings therefore may speak most directly to relatively senior teachers within the Greek education system.

### Randomized survey experiment design

Each teacher who completed the survey was randomly exposed to one of the four vignettes though computerized randomization in Qualtrics. Immediately after reading their assigned vignette, participants entered the grade they would award (0–10 scale). Based on this, we calculated the grading fairness gap—the main outcome of our study—which we define as the absolute deviation between the teacher’s grade and the objective or fair grade. Teachers then evaluated the grader on five dimensions—intent, ability, comprehension, responsibility, and fairness—using a Likert scale from −5 (“completely disagree”) to +5 (“completely agree”). Placing the behavioral outcome (the teacher’s grade) on a dedicated screen before presenting these attitudinal mediators shields the grading decision from evaluative priming and anchoring. This ordering accords with survey methodology guidance to keep outcome measures “uncontaminated” by attitudinal prompts (50, 51) and follows the order-effect warnings of Chaudoin et al. (52). We also collected a comprehensive set of demographic characteristics from respondents, including gender, age, teaching experience, subject specialization, location, and technological literacy. At the end of the experiment, we collected information on teachers’ own use of generative AI tools and their views on algorithmic grading. See Appendix S2 for a comprehensive list of all survey questions in the sequential order they were asked.

### Empirical strategy

We investigate the impact of the survey experiment by estimating the average effect of exposure to algorithmic vs. human grading recommendations separately for the harsh and lenient scenarios. To do so, we estimate the following model in each subsample:


(1) $$Y_{i}=\beta_{0}+\beta_{1}\cdot {\text{AI}}_{i}+X_{i}^{\prime }\gamma +\theta +\phi +\varepsilon_{i},$$

where $Y_{i}$ denotes the outcome variable—the absolute grading fairness gap—measured after participant *i* is exposed to a specific evaluation scenario. The binary variable ${\text{AI}}_{i}$ equals one if participant *i* was randomly assigned to receive an algorithmic grade recommendation and zero if the recommendation was attributed to a human grader. We include county fixed effects (*θ*) to account for spatial heterogeneity across participants’ locations and fixed effects for the teacher’s field of specialization (*ϕ*) to control for subject-specific grading patterns. Teachers graded exercises that matched their subject area of expertise (ie Mathematics, Ancient Greek, Modern Greek, or Science). Vector $X_{i}$ captures participant characteristics, with indicators included for any missing values in the covariates. Participant controls include gender, age, educational attainment, teaching experience, years since graduation, an indicator for whether the participant studied abroad, and a baseline measure of grading leniency.

The coefficient $\beta_{1}$ represents the effect of algorithmic (vs. human) recommendations on the grading fairness gap within each scenario. We estimate Eq. (1) separately for the harsh and lenient grading conditions using ordinary least squares, with heteroskedasticity-robust standard errors clustered at county level. Because the recommendation source, ${\text{AI}}_{i}$, is randomly assigned, the coefficient $\beta_{1}$ can be interpreted as the conditional average causal effect of receiving an algorithmic (rather than human) recommendation on the grading-fairness gap, holding constant the covariates $X_{i}$, county fixed effects *θ*, and subject fixed effects *ϕ* within each grading scenario.

### Mediation analysis

We analyze how the source of an erroneous initial grade, AI grader vs. human colleague, influences teachers’ final grading decisions based on five hypothesized psychological mediators. Following Baron et al. (53), the total treatment effect is decomposed into indirect effects, with each tracing the portion of the intervention that operates through one mediator (*ability*, *comprehension*, *fairness*, *intent*, or *responsibility*), and a residual direct effect that captures any influence not explained by these channels.

For example, if teachers view the AI system as more technically competent, perceived *ability* may transmit part of the treatment effect and nudge teachers to align more closely with the algorithmic recommendation. Conversely, if human graders are judged to better understand student context, higher perceived *comprehension* could lead teachers to trust a colleague’s advice and discount the AI’s. The estimated indirect effects quantify the contribution of each perception, whereas the direct effect reflects any remaining impact of the grade’s source beyond these mediation pathways.

## Supplementary Material

pgag146_Supplementary_Data

## Data Availability

Anonymized participant-level data have been deposited in openICPSR at https://doi.org/10.3886/E247246V1 (54).

## Notes

a Figures S4–S6 also present distributions of teachers’ baseline technological literacy, age, and experience.

b Grading humanities-related tasks may involve more subjective judgment, leaving greater scope for deference to recommendations than grading STEM-related tasks.

c We also present the distribution of these variables in Fig. S9.

## Preprints

This manuscript was posted on a preprint: https://papers.ssrn.com/sol3/papers.cfm?abstract_id=5294732.
