# Supplementary material for: Why do experts miss AI’s errors? Evidence from a randomized labeling experiment
Source: PNAS Nexus. 2026 Jun 9;5(6):pgag146. doi: 10.1093/pnasnexus/pgag146 (PMC13248211; doi:10.1093/pnasnexus/pgag146)
Supplement: pgag146_Supplementary_Data [file pgag146_supplementary_data.pdf]

Supplementary Appendix

Figure S1: Respondent Density

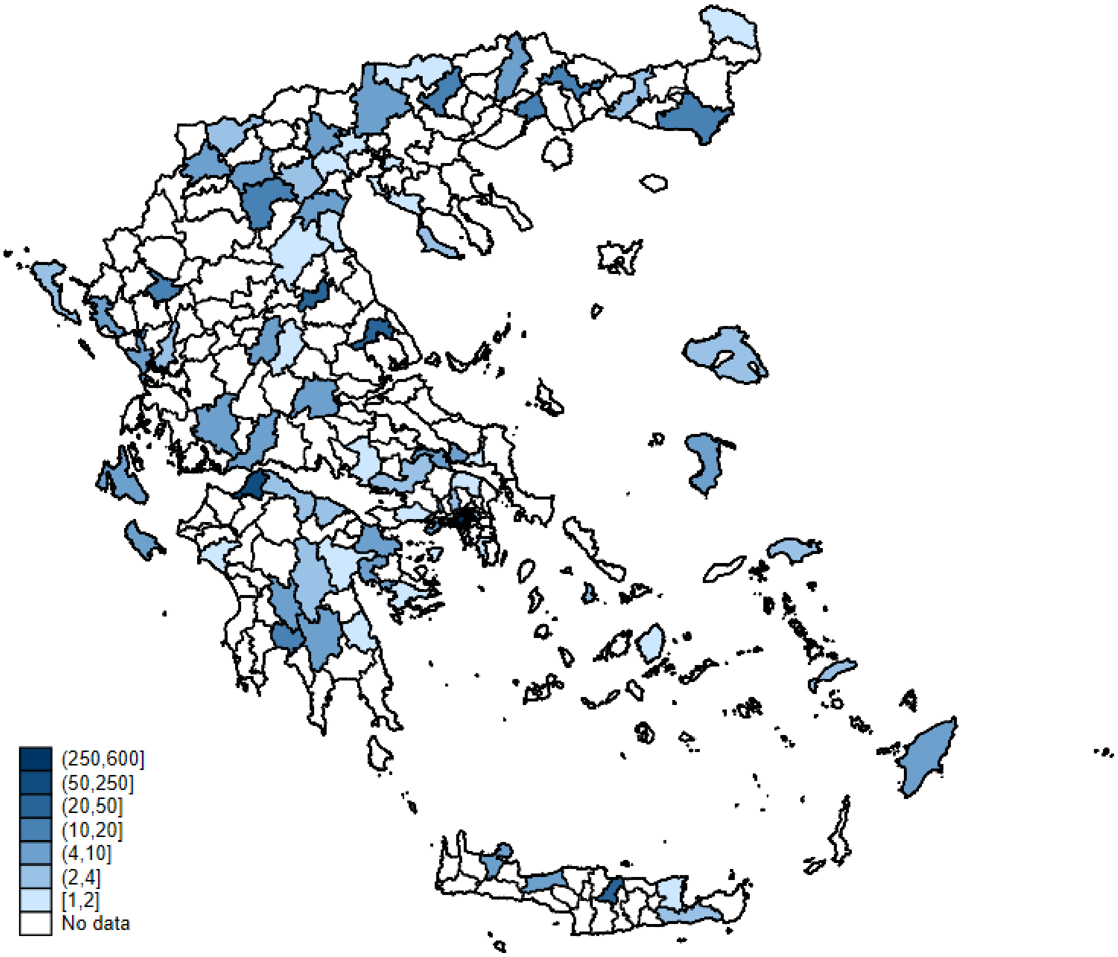

Notes: The map shows respondent density by prefecture across Greece.

Figure S2: Grade Distribution Across Treatment Conditions

Panel A: Harsh

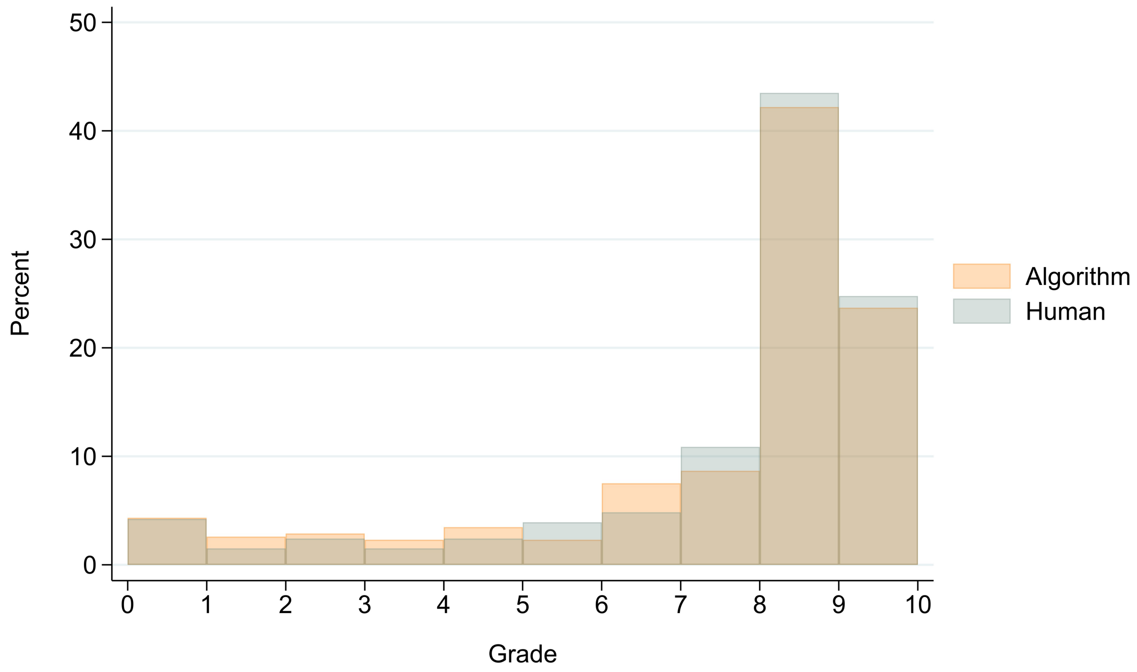

Panel B: Lenient

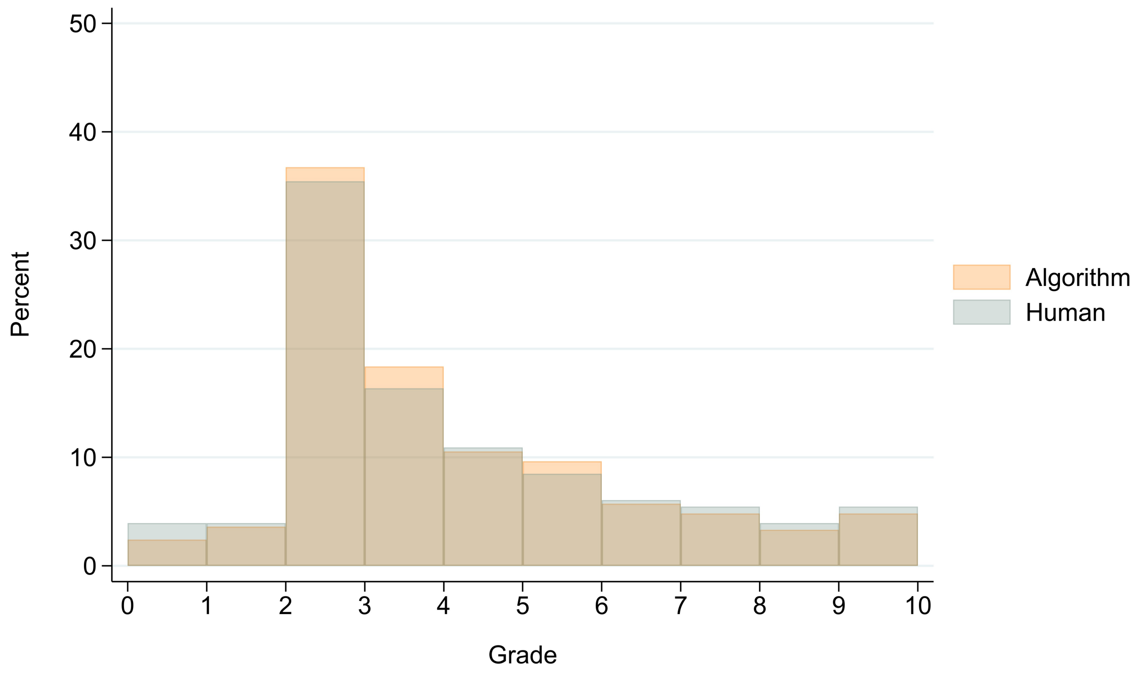

Notes: Panel A presents the distribution of grades across the *Human* and *Algorithm* treatment conditions under the scenario that involves a harsh recommender, and Panel B presents the corresponding distributions under a lenient recommender.

Figure S3: Histograms and Scatterplot of Grades and Grading Fairness Gap

Panel A: Harsh

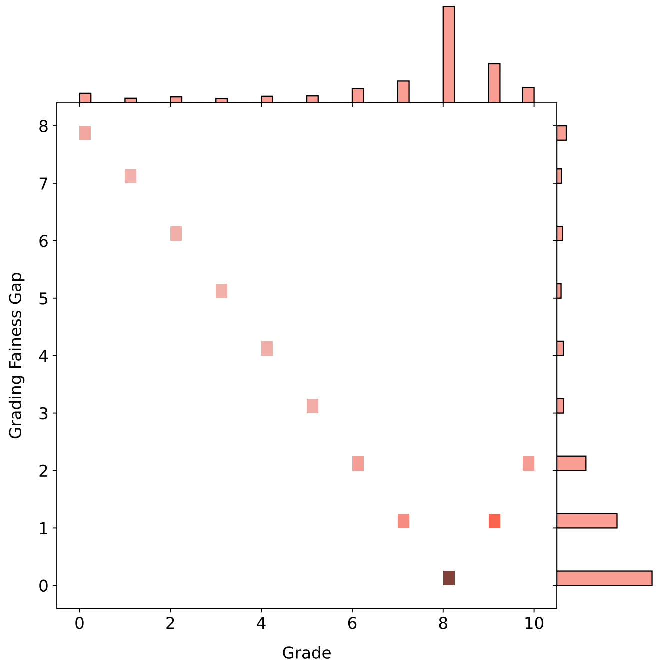

Panel B: Lenient

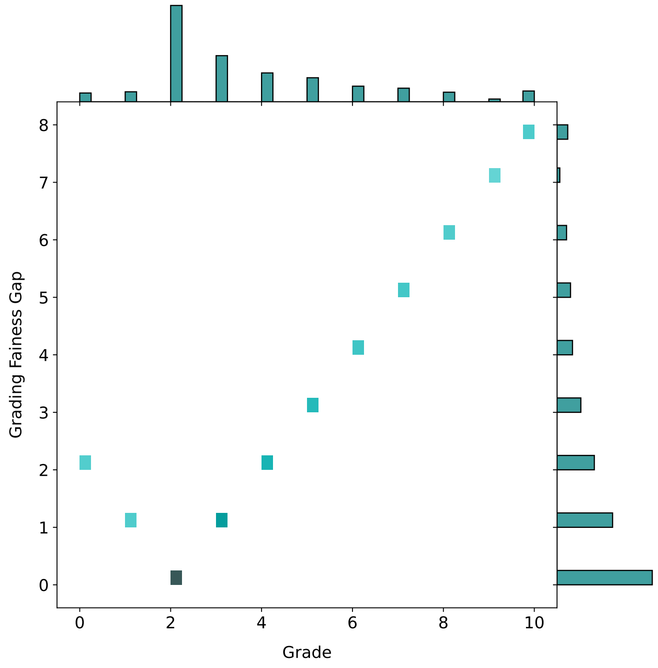

Notes: Grading fairness gap is the absolute difference between a teacher's grade and an objective or benchmark grade, used to measure the extent of deviation from fair grading. Grade refers to the grades teachers provided on the randomized survey instrument. Darker shades represent higher concentration of observations. Panels A and B correspond to the Harsh and Lenient scenario, respectively.

Figure S4: Distribution of Baseline Technological Literacy

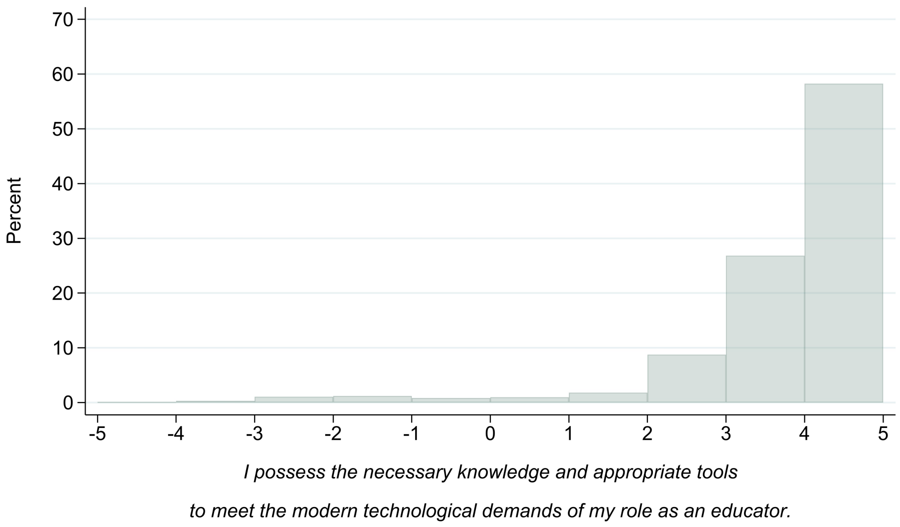

Notes: This figure shows the histogram of the self-reported baseline technological literacy score. *Tech Literacy* is measured on a scale from -5 (Completely Disagree) to 5 (Completely Agree), based on teachers' agreement with the statement: *"I possess the necessary knowledge and appropriate tools to meet the modern technological demands of my role as an educator."* Median *Baseline Technological Literacy* score is 4.

Figure S5: Distribution of Teachers' Age

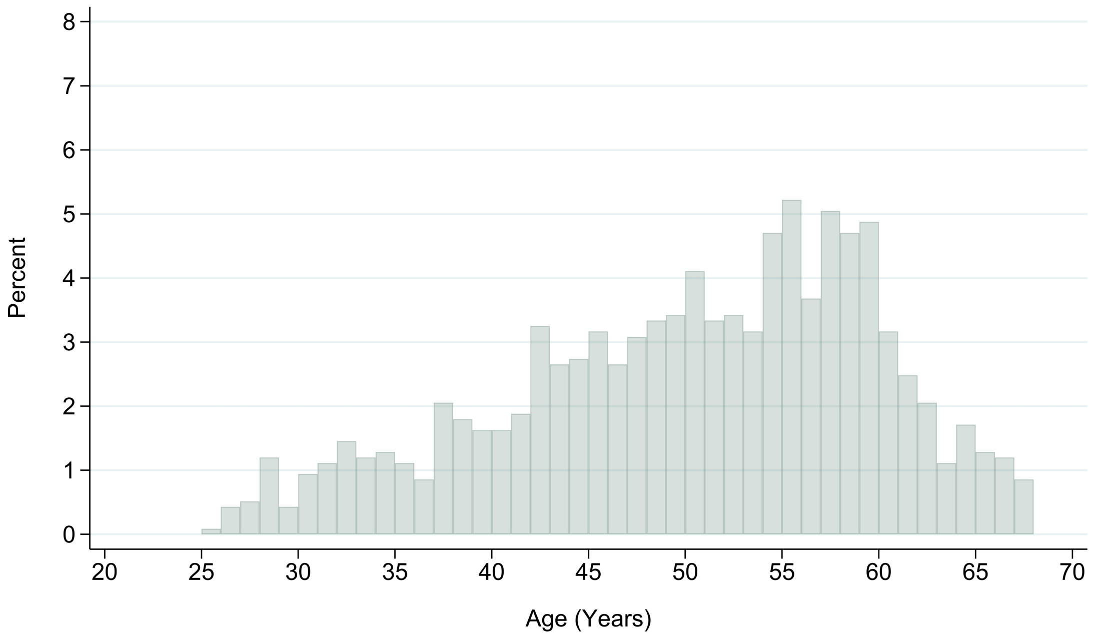

Notes: The figure shows the distribution of teachers' ages (in years). Median teacher age is 51 years.

Figure S6: Distribution of Teachers' Experience

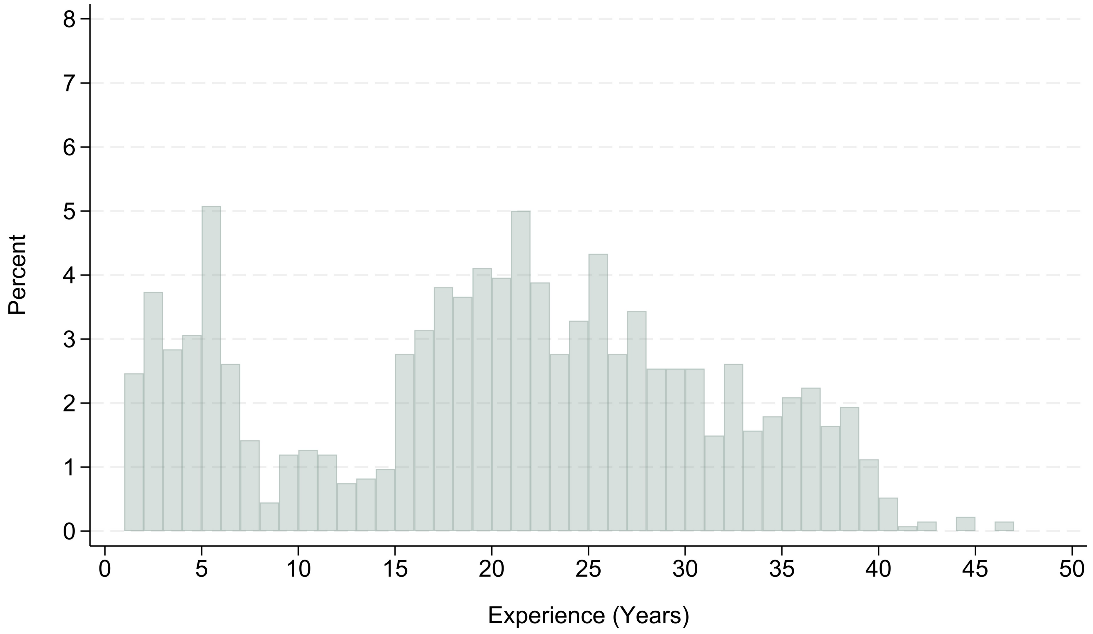

Notes: The figure shows the distribution of teachers' experience (in years). Median teacher experience is 21 years.

Figure S7: Differences in Perceived Mediators between Human and Algorithmic Recommenders

Panel A: Harsh

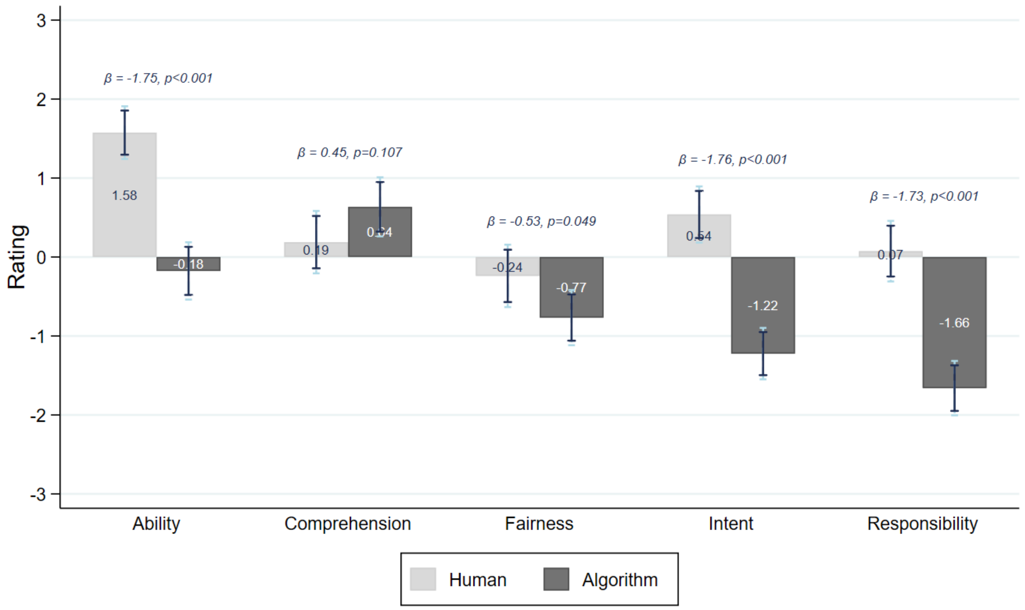

Panel B: Lenient

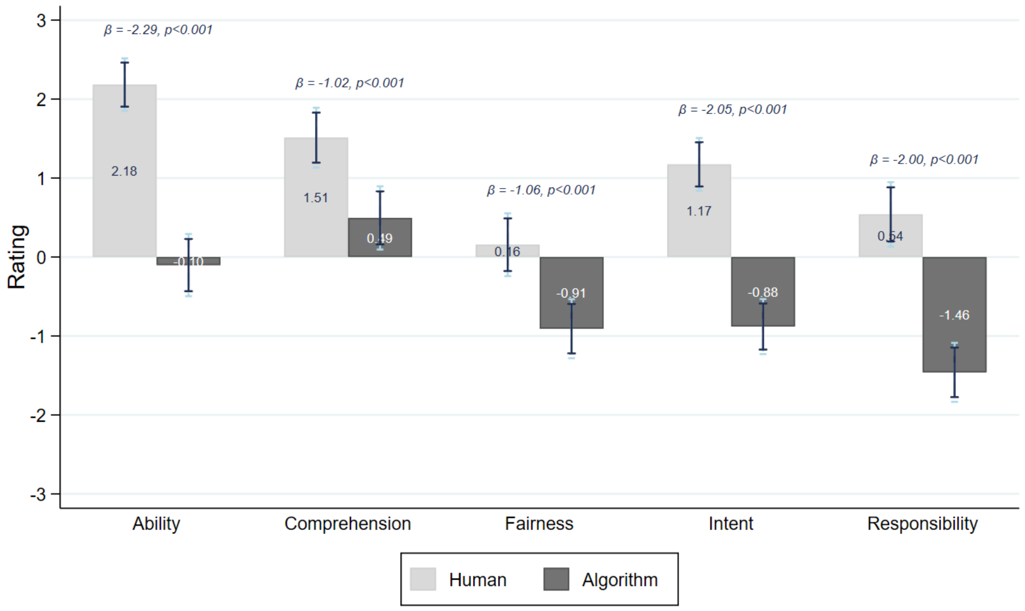

Notes: Panel A presents the mean differences in teachers' perceptions of mediators across the *Human* and *Algorithm* treatment conditions in the harsh recommender scenario and Panel B reports the same comparisons for the lenient recommender scenario. β represents mean differences, and p values indicate statistical significance based on two-sample mean comparison t tests. Dashed and solid error bars represent 90% and 95% confidence intervals, respectively.

Figure S8: Supplementary Differential Results by Teachers' Characteristics

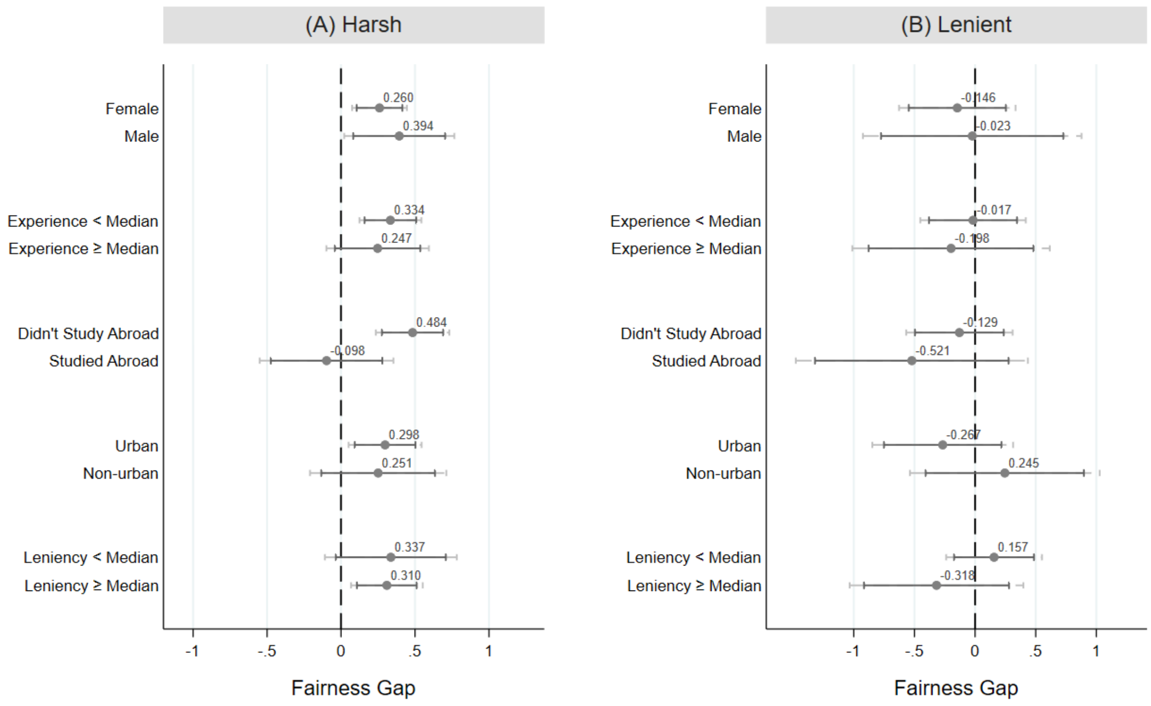

Notes: This figure plots the estimated treatment effect (algorithm vs. human) on the for teacher subgroups defined by gender, years of experience, overseas study, school locale, and baseline grading leniency. Positive coefficients indicate that the algorithmic recommendation increased the gap relative to the human recommendation; negative coefficients indicate that it reduced the gap. Panel (A) displays results for the harsh scenario and Panel (B) for the lenient scenario. *Grading fairness gap* is the absolute difference between a teacher's grade and an objective or benchmark grade and is used to measure the extent of deviation from fair grading. All specifications include a comprehensive set of control variables, such as gender, age, level of education, teaching experience, years since graduation, an indicator for whether the participant studied abroad, baseline grading leniency, and indicators to reflect any missing values. We also control for teachers' field of specialization and include county fixed effects. Standard errors are clustered at county level. Dashed and solid error bars represent 90% and 95% confidence intervals, respectively. Median experience is 21 years. Median leniency score is 7.

Figure S9: Teacher Attitudes Toward the Use of Artificial Intelligence in Grading

Panel A: Perceived Fairness of AI Grading

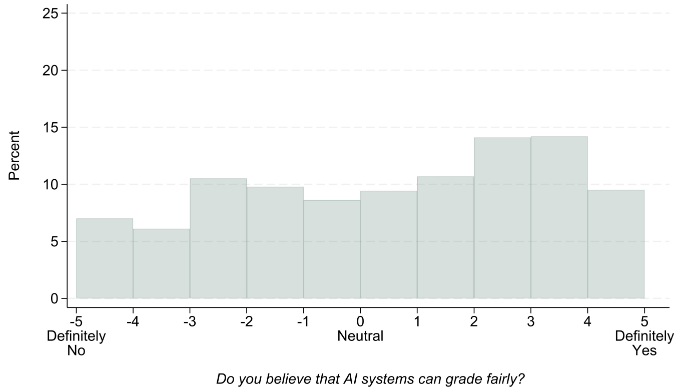

Panel B: Willingness to Use AI for Grading

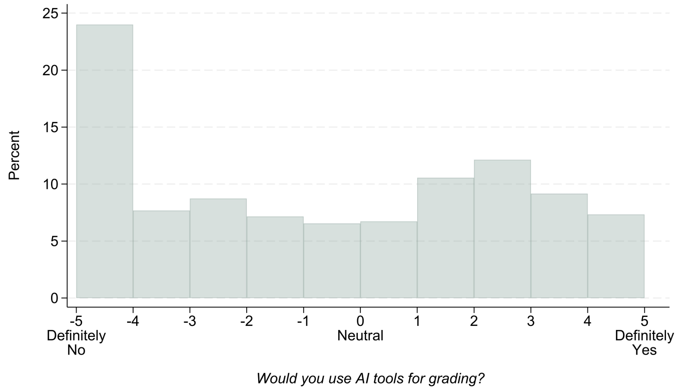

Panel C: Ethical Views on AI-Based Grading

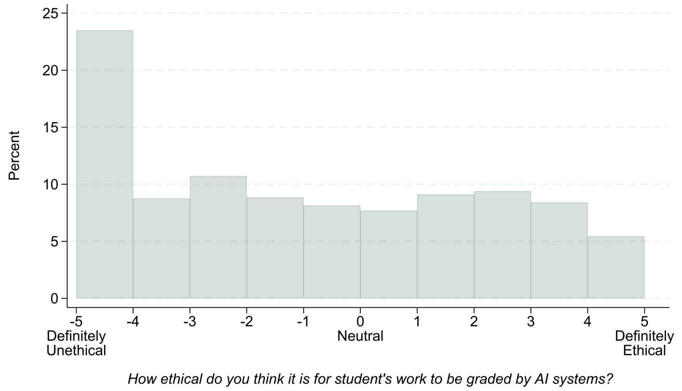

Notes: Panel A shows the distribution of teacher responses to whether they believe AI systems can grade fairly. Panel B displays teacher self-reported willingness to use AI tools for grading tasks. Panel C shows how ethical teachers perceive it is for student work to be graded by AI systems.

Table S1: Balance Tests between Harsh and Lenient Scenarios

	(1)	(2)	(3)	(4)	(5)	(6)	(7)	(8)
	Harsh			Lenient				
	Mean	SD	*N*	Mean	SD	*N*	Diff.	*P* value
Age (Yrs)	50.2	9.4	606	49.3	9.6	563	-0.924	0.097
Experience (Yrs)	20.3	10.8	677	19.3	10.8	662	-0.978	0.098
Years Since Graduation (Yrs)	27.4	10.1	677	26.7	10.0	662	-0.728	0.186
Baseline Leniency (0-10)	6.6	2.2	668	6.6	2.3	654	-0.015	0.903
	%		*N*	%		*N*	Diff.	*P* value
Gender								
Female	70.2		677	68.7		662	-1.431	0.570
Male	29.8		677	31.3		662	1.431	0.570
Education								
Bachelors	30.6		677	31.1		662	0.542	0.830
Masters	60.4		677	62.1		662	1.671	0.531
Doctorate	8.7		677	6.8		662	-1.917	0.190
Studied Abroad	23.7		604	22.4		562	-1.256	0.611
Field								
Humanities	54.8		677	51.8		662	-2.988	0.274
STEM	22.3		677	26.0		662	3.678	0.116
Primary/Other	22.9		677	22.2		662	-0.690	0.763
**Baseline Technological Literacy**								
"I possess the necessary knowledge and appropriate tools to meet the modern technological demands of my role as an educator."								
	Mean	SD	*N*	Mean	SD	*N*	Diff.	*P* value
Score	3.5	1.5	677	3.4	1.6	662	-0.101	0.220

Notes: The table reports summary statistics for teachers across the *Harsh* and *Lenient* scenarios. *Harsh* refers to the scenario in which a participant was exposed to a harsh recommender. *Lenient* refers to a treatment condition in which a participant was exposed to a lenient recommender. Field denotes the teacher's area of specialization and is categorized as follows: STEM, which encompasses subjects such as mathematics, physics, chemistry, science, and computer science; Humanities, which includes disciplines such as theology, art, sociology, and economics; and Primary/Other, which refers to Greek language instruction, primary education, or teaching in technical institutions. P values stemming from a two-sample mean comparison t test are reported. Leniency is assessed on a scale from 0 to 10 based on the grades assigned by participants in the baseline exercises. Self-reported *Baseline Technological Literacy* is measured on a scale from -5 (Completely Disagree) to 5 (Completely Agree). Figure S4 plots the distribution of the *Baseline Technological Literacy* measure.

Table S2: Summary Statistics for Mediators

	Scenario							
	Harsh		Lenient					
	Human	Algorithm	Human	Algorithm	P values from Two-sample T-tests			
**Mediator**	(1)	(2)	(3)	(4)	(1)-(2)	(3)-(4)	(1)-(3)	(2)-(4)
Ability	1.576	-0.176	2.183	-0.102	<0.001	<0.001	0.011	0.787
	(2.920)	(3.289)	(2.874)	(3.428)				
Comprehension	0.189	0.635	1.512	0.495	0.107	<0.001	<0.001	0.615
	(3.460)	(3.392)	(3.264)	(3.482)				
Fairness	-0.239	-0.767	0.156	-0.908	0.049	<0.001	0.166	0.589
	(3.461)	(3.167)	(3.434)	(3.250)				
Intent	0.539	-1.223	1.173	-0.881	<0.001	<0.001	0.011	0.158
	(3.110)	(2.950)	(2.883)	(3.029)				
Responsibility	0.074	-1.660	0.540	-1.461	<0.001	<0.001	0.103	0.440
	(3.359)	(3.122)	(3.532)	(3.249)				

Notes: This table reports summary statistics for the variables used as mediators in the analysis. For each mediator, we present the mean and standard deviation (in parentheses). Mediators are measured on a standardized scale ranging from –5 (Completely Disagree) to 5 (Completely Agree).

Table S3: Summary Statistics for Mediators by Teacher Specialization

	Teacher Specialization								
	STEM		Humanities		Primary/Other		P values from Two-sample T-tests		
	Human	Algorithm	Human	Algorithm	Human	Algorithm			
**Mediator**	(1)	(2)	(3)	(4)	(5)	(6)	(1)-(2)	(3)-(4)	(5)-(6)
Harsh Scenario									
Ability	1.525	0.757	1.755	-0.631	1.219	0.015	0.141	<0.001	0.015
	(2.913)	(3.105)	(3.027)	(3.385)	(2.678)	(3.018)			
Comprehension	-0.361	0.135	0.632	0.520	-0.342	1.523	0.393	0.767	0.001
	(3.483)	(3.168)	(3.437)	(3.576)	(3.396)	(2.974)			
Fairness	-0.738	-0.851	0.123	-0.810	-0.630	-0.554	0.842	0.010	0.894
	(3.492)	(3.028)	(3.440)	(3.199)	(3.434)	(3.269)			
Intent	-0.361	-1.095	1.037	-1.419	0.178	-0.831	0.165	<0.001	0.054
	(3.225)	(2.790)	(2.965)	(3.018)	(3.155)	(2.935)			
Responsibility	-0.475	-1.824	0.368	-1.765	-0.123	-1.185	0.015	<0.001	0.062
	(3.289)	(2.995)	(3.346)	(3.146)	(3.419)	(3.196)			
Lenient Scenario									
Ability	2.260	-0.667	1.675	-0.370	3.355	1.028	<0.001	<0.001	<0.001
	(2.544)	(3.379)	(3.164)	(3.436)	(2.049)	(3.246)			
Comprehension	1.274	-0.853	1.032	0.438	2.984	2.014	<0.001	0.137	0.041
	(3.159)	(3.447)	(3.435)	(3.458)	(2.466)	(2.967)			
Fairness	-0.562	-1.960	-0.156	-1.185	1.774	0.750	0.010	0.008	0.047
	(3.468)	(3.015)	(3.409)	(3.233)	(2.967)	(2.915)			
Intent	0.822	-1.320	0.825	-1.240	2.452	0.306	<0.001	<0.001	<0.001
	(2.705)	(2.641)	(3.040)	(3.189)	(2.302)	(2.792)			
Responsibility	-0.027	-2.240	0.143	-1.712	2.194	-0.139	<0.001	<0.001	<0.001
	(3.366)	(2.903)	(3.600)	(3.293)	(3.077)	(3.155)			

Notes: This table reports summary statistics for the mediator variables used in the analysis by teacher specialization and evaluator type (human vs. algorithm). For each mediator, we report the mean and standard deviation (in parentheses). The last three columns report p-values from two-sample t-tests comparing human and algorithm evaluations within each specialization group. Mediators are measured on a standardized scale ranging from –5 (Completely Disagree) to 5 (Completely Agree).

Table S4: Summary Statistics for Mediators by Teacher Age

	Teacher Age							
	Below Median		Above Median					
	Human	Algorithm	Human	Algorithm	Two-sample T-tests			
**Mediator**	(1)	(2)	(3)	(4)	(1)-(2)	P-value	(3)-(4)	P-value
				Harsh Scenario				
Ability	1.938	-0.490	1.333	0.106	2.428	<0.001	1.227	0.001
	(2.685)	(3.049)	(3.091)	(3.491)				
Comprehension	0.359	0.587	0.133	0.712	-0.228	0.574	-0.579	0.133
	(3.506)	(3.125)	(3.406)	(3.628)				
Fairness	0.031	-0.853	-0.394	-0.659	0.884	0.023	0.265	0.483
	(3.358)	(2.950)	(3.526)	(3.369)				
Intent	0.883	-1.196	0.315	-1.247	2.079	<0.001	1.562	<0.001
	(2.910)	(2.657)	(3.236)	(3.212)				
Responsibility	0.359	-1.881	-0.103	-1.476	2.240	<0.001	1.373	<0.001
	(3.250)	(2.881)	(3.448)	(3.341)				
				Lenient Scenario				
Ability	2.105	-0.592	2.248	0.397	2.697	<0.001	1.851	<0.001
	(2.586)	(3.153)	(3.199)	(3.601)				
Comprehension	1.098	0.109	1.839	0.816	0.989	0.012	1.023	0.015
	(3.242)	(3.398)	(3.310)	(3.559)				
Fairness	-0.203	-1.102	0.547	-0.824	0.899	0.016	1.371	0.001
	(3.305)	(3.009)	(3.540)	(3.451)				
Intent	1.112	-0.993	1.314	-0.875	2.105	<0.001	2.189	<0.001
	(2.753)	(2.875)	(3.004)	(3.175)				
Responsibility	0.392	-1.660	0.672	-1.375	2.052	<0.001	2.047	<0.001
	(3.342)	(2.983)	(3.728)	(3.455)				

Notes: This table reports summary statistics for the mediator variables used in the analysis by teacher age and evaluator type (human vs. algorithm). For each mediator, we report the mean and standard deviation (in parentheses). We also report differences and p-values from two-sample t-tests comparing human and algorithm evaluations within each subgroup. Mediators are measured on a standardized scale ranging from –5 (Completely Disagree) to 5 (Completely Agree).

Table S5: Summary Statistics for Mediators by Teacher Educational Attainment

	Teacher Educational Attainment							
	Bachelors		Masters/Doctorate					
	Human	Algorithm	Human	Algorithm	Two-sample T-tests			
**Mediator**	(1)	(2)	(3)	(4)	(1)-(2)	P-value	(3)-(4)	P-value
				Harsh Scenario				
Ability	1.385	-0.053	1.667	-0.229	1.438	0.002	1.896	<0.001
	(2.993)	(3.334)	(2.888)	(3.277)				
Comprehension	0.177	0.579	0.194	0.659	-0.402	0.417	-0.465	0.166
	(3.378)	(3.447)	(3.507)	(3.376)				
Fairness	-0.063	-0.589	-0.323	-0.843	0.526	0.287	0.520	0.105
	(3.488)	(3.328)	(3.454)	(3.100)				
Intent	0.708	-1.326	0.458	-1.179	2.034	<0.001	1.637	<0.001
	(3.185)	(2.980)	(3.079)	(2.943)				
Responsibility	-0.094	-1.305	0.154	-1.812	1.211	0.013	1.966	<0.001
	(3.431)	(3.223)	(3.330)	(3.073)				
				Lenient Scenario				
Ability	1.926	0.258	2.308	-0.260	1.668	0.001	2.568	<0.001
	(3.115)	(3.485)	(2.750)	(3.399)				
Comprehension	1.309	0.584	1.610	0.456	0.725	0.142	1.154	0.001
	(3.405)	(3.243)	(3.198)	(3.589)				
Fairness	0.043	-0.719	0.210	-0.990	0.762	0.117	1.200	<0.001
	(3.442)	(3.093)	(3.437)	(3.320)				
Intent	0.883	-0.596	1.313	-1.005	1.479	0.001	2.318	<0.001
	(2.943)	(2.895)	(2.850)	(3.084)				
Responsibility	0.298	-1.135	0.656	-1.603	1.433	0.006	2.259	<0.001
	(3.654)	(3.382)	(3.476)	(3.188)				

Notes: This table reports summary statistics for the mediator variables used in the analysis by teacher educational attainment and evaluator type (human vs. algorithm). For each mediator, we report the mean and standard deviation (in parentheses). We also report differences and p-values from two-sample t-tests comparing human and algorithm evaluations within each subgroup. Mediators are measured on a standardized scale ranging from –5 (Completely Disagree) to 5 (Completely Agree).

Table S6: Summary Statistics for Mediators by Teacher Technological Literacy

	Teacher Technological Literacy							
	Below Median		Above Median					
	Human	Algorithm	Human	Algorithm	Two-sample T-tests			
**Mediator**	(1)	(2)	(3)	(4)	(1)-(2)	P-value	(3)-(4)	P-value
				Harsh Scenario				
Ability	1.069	-0.380	1.901	-0.037	1.449	<0.001	1.938	<0.001
	(2.906)	(3.217)	(2.891)	(3.340)				
Comprehension	0.181	0.496	0.193	0.730	-0.315	0.451	-0.537	0.145
	(3.235)	(3.293)	(3.606)	(3.464)				
Fairness	-0.440	-0.946	-0.110	-0.646	0.506	0.229	0.536	0.126
	(3.389)	(3.158)	(3.510)	(3.175)				
Intent	0.595	-1.225	0.503	-1.222	1.820	<0.001	1.725	<0.001
	(3.140)	(2.892)	(3.100)	(2.997)				
Responsibility	-0.267	-1.822	0.293	-1.550	1.555	<0.001	1.843	<0.001
	(3.361)	(3.058)	(3.350)	(3.168)				
				Lenient Scenario				
Ability	2.000	0.187	2.314	-0.346	1.813	<0.001	2.660	<0.001
	(2.759)	(3.403)	(2.954)	(3.440)				
Comprehension	1.250	0.284	1.698	0.673	0.966	0.017	1.025	0.009
	(3.093)	(3.306)	(3.377)	(3.626)				
Fairness	0.183	-0.754	0.136	-1.038	0.937	0.022	1.174	0.002
	(3.318)	(3.120)	(3.524)	(3.360)				
Intent	0.875	-0.948	1.385	-0.824	1.823	<0.001	2.209	<0.001
	(2.770)	(2.843)	(2.950)	(3.185)				
Responsibility	0.458	-1.545	0.598	-1.390	2.003	<0.001	1.988	<0.001
	(3.370)	(3.118)	(3.652)	(3.364)				

Notes: This table reports summary statistics for the mediator variables used in the analysis by teacher technological literacy and evaluator type (human vs. algorithm). For each mediator, we report the mean and standard deviation (in parentheses). We also report differences and p-values from two-sample t-tests comparing human and algorithm evaluations within each subgroup. Mediators are measured on a standardized scale ranging from -5 (Completely Disagree) to 5 (Completely Agree).

Table S7: Algorithm × Harsh Scenario Interaction Effects on Grading Fairness Gap

	Grading Fairness Gap	
	(1)	(2)
Algorithm × Harsh Scenario	0.355**	0.417**
	(0.157)	(0.167)
Algorithm	-0.089	-0.095
	(0.238)	(0.225)
Harsh Scenario	-0.662***	-0.701***
	(0.136)	(0.127)
Observations	1,339	1,339
Controls	No	Yes

Notes: This table reports estimates from a multiple regression specification. *Grading fairness gap* is the absolute difference between a teacher's grade and an objective or benchmark grade, and is used to measure the extent of deviation from fair grading. All specifications include teacher field of specialization and county fixed effects. Specifications with controls include a comprehensive set of control variables, such as gender, age, level of education, teaching experience, years since graduation, an indicator for whether the participant studied abroad, baseline grading leniency, and indicators to reflect any missing values. Standard errors clustered at county level are reported in parentheses. * $p < 0.1$; ** $p < 0.05$; *** $p < 0.01$.

Table S8: ROBUSTNESS ANALYSIS: FAIRNESS GAP WITHOUT ABSOLUTE VALUE

	(1)	(2)	(3)	(4)	(5)	(6)	(7)
	Means		Without Controls		With Controls		
Scenario	Human	Algorithm	$\hat{\beta}$	SE	$\hat{\beta}$	SE	N
Harsh	0.785	0.942	0.247***	0.083	0.260***	0.082	677
Lenient	-1.694	-1.645	0.054	0.282	0.002	0.288	662

Notes: Parameter $\hat{\beta}$ is the estimated parameter of interest from a multiple regression specification. *Grading fairness gap* is the difference between a teacher's grade and the fair grade. This gap measures the extent of deviation from fair grading. All specifications include teacher field of specialization and county fixed effects. Specifications with controls include a comprehensive set of control variables, such as gender, age, level of education, teaching experience, years since graduation, an indicator for whether the participant studied abroad, baseline grading leniency, and indicators to reflect any missing values. Standard errors clustered at county level are reported in parentheses. *, ** and *** indicate statistical significance at the 10%, 5%, and 1% level, respectively.

Table S9: Robustness Analysis: Exclusion of Out-of-Range Grades

	(1)	(2)	(3)	(4)	(5)	(6)	(7)
	Means		Without Controls		With Controls		
Scenario	Human	Algorithm	$\hat{\beta}$	SE	$\hat{\beta}$	SE	N
Harsh	1.442	1.655	0.450***	0.106	0.483***	0.110	513
Lenient	1.967	1.840	-0.098	0.285	-0.092	0.268	616

Notes: Parameter $\hat{\beta}$ is the estimated parameter of interest from a multiple regression specification. *Grading fairness gap* is the absolute difference between a teacher's grade and an objective or benchmark grade, and is used to measure the extent of deviation from fair grading. All specifications include teacher field of specialization and county fixed effects. Specifications with controls include a comprehensive set of control variables, such as gender, age, level of education, teaching experience, years since graduation, an indicator for whether the participant studied abroad, baseline grading leniency, and indicators to reflect any missing values. Standard errors clustered at county level are reported in parentheses. *, ** and *** indicate statistical significance at the 10%, 5% and 1% level, respectively.

Table S10: Robustness Analysis: Alternative Definitions of Fair Grade

	(1)	(2)	(3)	(4)	(5)	(6)	(7)
	Means		Without Controls		With Controls		
Scenario	Human	Algorithm	$\hat{\beta}$	SE	$\hat{\beta}$	SE	N
Panel A: Fair Grade + 1							
Harsh	1.892	2.107	0.299***	0.094	0.318***	0.086	679
Lenient	1.796	1.669	-0.129	0.210	-0.134	0.198	665
Panel B: Fair Grade - 1							
Harsh	1.749	1.902	0.207*	0.118	0.236**	0.106	668
Lenient	2.773	2.693	-0.081	0.265	-0.051	0.280	652

Notes: Parameter $\hat{\beta}$ is the estimated parameter of interest from a multiple regression specification. *Grading fairness gap* is the absolute difference between a teacher's grade and an objective or benchmark grade, and is used to measure the extent of deviation from fair grading. All specifications include teacher field of specialization and county fixed effects. Specifications with controls include a comprehensive set of control variables, such as gender, age, level of education, teaching experience, years since graduation, an indicator for whether the participant studied abroad, baseline grading leniency, and indicators to reflect any missing values. Standard errors clustered at county level are reported in parentheses. *, ** and *** indicate statistical significance at the 10%, 5%, and 1% level, respectively.

Table S11: Cronbach's Alpha Coefficients for Mediator Combinations

	Scenario	
Mediator Combination	Harsh	Lenient
Ability, Comprehension	0.659	0.749
Ability, Fairness	0.794	0.799
Ability, Intent	0.724	0.737
Ability, Responsibility	0.813	0.839
Comprehension, Fairness	0.816	0.802
Comprehension, Intent	0.666	0.664
Comprehension, Responsibility	0.745	0.818
Fairness, Intent	0.828	0.781
Fairness, Responsibility	0.888	0.910
Intent, Responsibility	0.832	0.807
Ability, Comprehension, Fairness	0.826	0.845
Ability, Comprehension, Intent	0.763	0.793
Ability, Comprehension, Responsibility	0.812	0.860
Ability, Fairness, Intent	0.845	0.837
Ability, Fairness, Responsibility	0.883	0.896
Ability, Intent, Responsibility	0.851	0.856
Comprehension, Fairness, Intent	0.837	0.820
Comprehension, Fairness, Responsibility	0.871	0.892
Comprehension, Intent, Responsibility	0.819	0.834
Fairness, Intent, Responsibility	0.895	0.886
Ability, Comprehension, Fairness, Intent	0.858	0.862
Ability, Comprehension, Fairness, Responsibility	0.883	0.903
Ability, Comprehension, Intent, Responsibility	0.853	0.873
Ability, Fairness, Intent, Responsibility	0.899	0.899
Comprehension, Fairness, Intent, Responsibility	0.889	0.891
Ability, Comprehension, Fairness, Intent, Responsibility	0.899	0.907

Notes: This table reports Cronbach's alpha coefficients for different combinations of mediators across the two scenarios.

Table S12: Impact of Algorithmic Recommender on Perceived Mediators

	(1)	(2)	(3)	(4)	(5)
	Outcome Variable				
	Ability	Comprehension	Fairness	Intent	Responsibility
	Harsh Recommender				
Algorithm (Vs. Human)	-1.779***	0.379	-0.495	-1.687***	-1.714***
	(0.172)	(0.350)	(0.331)	(0.196)	(0.270)
N	615	615	615	615	615
	Lenient Recommender				
Algorithm (Vs. Human)	-2.247***	-0.823*	-0.884*	-1.909***	-1.841***
	(0.230)	(0.426)	(0.470)	(0.456)	(0.498)
N	582	582	582	582	582

Notes: This table presents the effects of the algorithmic recommender compared with the human recommender on perceived mediators. Mediators are measured on a standardized scale ranging from –5 (Completely Disagree) to 5 (Completely Agree). All specifications include a comprehensive set of control variables, such as gender, age, level of education, teaching experience, years since graduation, an indicator for whether the participant studied abroad, baseline grading leniency, and indicators to reflect any missing values. We also control for teachers' fields of specialization and include county fixed effects. Standard errors clustered at county level are reported in parentheses. *, ** and *** indicate statistical significance at the 10%, 5%, and 1% level, respectively.

Table S13: ALGORITHM × SCENARIO INTERACTION EFFECTS ON PERCEIVED MEDIATORS

	(1)	(2)	(3)	(4)	(5)
	Outcome Variable				
	Ability	Comprehension	Fairness	Intent	Responsibility
Algorithm × Harsh Scenario	0.560**	1.323**	0.477	0.265	0.196
	(0.268)	(0.608)	(0.565)	(0.409)	(0.594)
Algorithm	-2.270***	-0.890**	-0.949**	-1.961***	-1.903***
	(0.207)	(0.357)	(0.387)	(0.326)	(0.413)
Harsh Scenario	-0.512***	-1.221***	-0.268	-0.538***	-0.290
	(0.179)	(0.282)	(0.267)	(0.181)	(0.339)
N	1,197	1,197	1,197	1,197	1,197

Notes: This table reports estimates from a multiple regression specification. Mediators are measured on a standardized scale ranging from –5 (Completely Disagree) to 5 (Completely Agree). All specifications include a comprehensive set of control variables, such as gender, age, level of education, teaching experience, years since graduation, an indicator for whether the participant studied abroad, baseline grading leniency, and indicators to reflect any missing values. We also control for teachers' field of specialization and include county fixed effects. Standard errors clustered at county level are reported in parentheses. * $p < 0.1$; ** $p < 0.05$; *** $p < 0.01$.

Table S14: Mediated Grading Fairness Gap, Sequential Inclusion of Mediators, Harsh Scenario

	Outcome: Grading Fairness Gap						
	(1)	(2)	(3)	(4)	(5)	(6)	(7)
Algorithm (Vs. Human)	0.342**	0.161	0.346***	0.327**	0.311*	0.260*	0.072
	(0.128)	(0.129)	(0.125)	(0.129)	(0.167)	(0.147)	(0.165)
**Mediators:**							
Ability		-0.102***					-0.146***
		(0.014)					(0.030)
Comprehension			-0.009				0.044
			(0.014)				(0.026)
Fairness				-0.031			0.037
				(0.022)			(0.039)
Intent					-0.018		0.028
					(0.036)		(0.049)
Responsibility						-0.048**	-0.035
						(0.019)	(0.024)
N	606	606	606	606	606	606	606
Participant Controls	✓	✓	✓	✓	✓	✓	✓

Notes: This table presents the effects of the algorithmic recommender compared with the human recommender on the , controlling for perceived mediators, within the harsh scenario. *Grading fairness gap* is the absolute difference between a teacher's grade and an objective or benchmark grade, and is used to measure the extent of deviation from fair grading. All specifications include a comprehensive set of control variables, such as gender, age, level of education, teaching experience, years since graduation, an indicator for whether the participant studied abroad, baseline grading leniency, and indicators to reflect any missing values. We also control for teachers' field of specialization and include county fixed effects. Standard errors clustered at county level are reported in parentheses. *, ** and *** indicate statistical significance at the 10%, 5%, and 1% level, respectively.

Table S15: Mediated Grading Fairness Gap, Sequential Exclusion of Mediators, Harsh Scenario

	Outcome: Grading Fairness Gap						
	(1)	(2)	(3)	(4)	(5)	(6)	(7)
Algorithm (Vs. Human)	0.342**	0.100	0.044	0.101	0.126	0.237	0.072
	(0.128)	(0.164)	(0.137)	(0.177)	(0.150)	(0.167)	(0.165)
**Mediators:**							
Ability		-0.152***	-0.145***	-0.139***	-0.141***		-0.146***
		(0.030)	(0.029)	(0.025)	(0.032)		(0.030)
Comprehension		0.041	0.045*	0.053**		0.030	0.044
		(0.027)	(0.024)	(0.020)		(0.026)	(0.026)
Fairness		0.021	0.047		0.059*	-0.010	0.037
		(0.039)	(0.048)		(0.029)	(0.032)	(0.039)
Intent		0.020		0.036	0.031	0.024	0.028
		(0.048)		(0.053)	(0.050)	(0.047)	(0.049)
Responsibility			-0.027	-0.020	-0.028	-0.076***	-0.035
			(0.021)	(0.027)	(0.025)	(0.025)	(0.024)
N	606	606	606	606	606	606	606
Participant Controls	✓	✓	✓	✓	✓	✓	✓

Notes: This table presents the effects of the algorithmic recommender compared with the human recommender on the , controlling for perceived mediators, within the harsh scenario. *Grading fairness gap* is the absolute difference between a teacher's grade and an objective or benchmark grade, and is used to measure the extent of deviation from fair grading. All specifications include a comprehensive set of control variables, such as gender, age, level of education, teaching experience, years since graduation, an indicator for whether the participant studied abroad, baseline grading leniency, and indicators to reflect any missing values. We also control for teachers' field of specialization and include county fixed effects. Standard errors clustered at county level are reported in parentheses. *, ** and *** indicate statistical significance at the 10%, 5%, and 1% level, respectively.

Table S16: Mediated Grading Fairness Gap, Sequential Inclusion of Mediators, Lenient Scenario

	Outcome: Grading Fairness Gap						
	(1)	(2)	(3)	(4)	(5)	(6)	(7)
Algorithm (Vs. Human)	-0.174	0.099	-0.037	0.009	0.114	0.171	0.013
	(0.224)	(0.230)	(0.187)	(0.187)	(0.233)	(0.183)	(0.214)
**Mediators:**							
Ability		0.122***					-0.058**
		(0.024)					(0.025)
Comprehension			0.166***				0.063**
			(0.021)				(0.027)
Fairness				0.207***			0.143***
				(0.022)			(0.019)
Intent					0.151***		0.012
					(0.035)		(0.042)
Responsibility						0.187***	0.064
						(0.024)	(0.049)
N	573	573	573	573	573	573	573
Participant Controls	✓	✓	✓	✓	✓	✓	✓

Notes: This table presents the effects of the algorithmic recommender compared with the human recommender on the , controlling for perceived mediators, within the lenient scenario. *Grading fairness gap* is the absolute difference between a teacher's grade and an objective or benchmark grade, and is used to measure the extent of deviation from fair grading. All specifications include a comprehensive set of control variables, such as gender, age, level of education, teaching experience, years since graduation, an indicator for whether the participant studied abroad, baseline grading leniency, and indicators to reflect any missing values. We also control for teachers' field of specialization and include county fixed effects. Standard errors clustered at county level are reported in parentheses. *, ** and *** indicate statistical significance at the 10%, 5%, and 1% level, respectively.

Table S17: Mediated Grading Fairness Gap, Sequential Exclusion of Mediators, Lenient Scenario

	Outcome: Grading Fairness Gap						
	(1)	(2)	(3)	(4)	(5)	(6)	(7)
Algorithm (Vs. Human)	-0.174	-0.017	0.002	0.124	0.038	0.081	0.013
	(0.224)	(0.228)	(0.185)	(0.211)	(0.220)	(0.204)	(0.214)
**Mediators:**							
Ability		-0.046**	-0.057**	-0.046*	-0.045		-0.058**
		(0.022)	(0.027)	(0.026)	(0.027)		(0.025)
Comprehension		0.073***	0.062**	0.082***		0.052*	0.063**
		(0.023)	(0.027)	(0.028)		(0.027)	(0.027)
Fairness		0.176***	0.146***		0.158***	0.135***	0.143***
		(0.027)	(0.022)		(0.019)	(0.018)	(0.019)
Intent		0.023		0.035	0.010	0.005	0.012
		(0.037)		(0.044)	(0.041)	(0.043)	(0.042)
Responsibility			0.067	0.145***	0.084*	0.045	0.064
			(0.042)	(0.047)	(0.048)	(0.047)	(0.049)
N	573	573	573	573	573	573	573
Participant Controls	✓	✓	✓	✓	✓	✓	✓

Notes: This table presents the effects of the algorithmic recommender compared with the human recommender on the , controlling for perceived mediators, within the lenient scenario. *Grading fairness gap* is the absolute difference between a teacher's grade and an objective or benchmark grade, and is used to measure the extent of deviation from fair grading. All specifications include a comprehensive set of control variables, such as gender, age, level of education, teaching experience, years since graduation, an indicator for whether the participant studied abroad, baseline grading leniency, and indicators to reflect any missing values. We also control for teachers' field of specialization and include county fixed effects. Standard errors clustered at county level are reported in parentheses. *, ** and *** indicate statistical significance at the 10%, 5%, and 1% level, respectively.

Table S18: DIFFERENTIAL RESULTS BY TEACHERS' GENDER, AGE, EDUCATION, EXPERIENCE, AND LOCALE

	(1)	(2)	(3)	(4)	(5)	(6)	(7)
	Means		Without Controls		With Controls		
Scenario	Human	Algorithm	$\hat{\beta}$	SE	$\hat{\beta}$	SE	N
**By Gender**							
Harsh							
Male	1.153	1.385	0.357*	0.195	0.394**	0.185	202
Female	1.481	1.669	0.224**	0.108	0.260***	0.092	475
Lenient							
Male	1.944	1.929	0.047	0.433	-0.023	0.448	207
Female	1.923	1.764	-0.176	0.253	-0.146	0.239	455
**By Age**							
Harsh							
Above Median	1.600	1.724	0.218	0.238	0.251	0.211	335
Below Median	1.094	1.538	0.390***	0.088	0.463***	0.105	271
Lenient							
Above Median	2.036	1.728	-0.341	0.283	-0.360	0.305	273
Below Median	1.776	1.680	-0.042	0.286	-0.081	0.245	290
**By Education**							
Harsh							
Masters/Doctorat	1.281	1.545	0.359***	0.120	0.442***	0.093	468
Bachelors	1.598	1.676	0.076	0.167	-0.022	0.213	209
Lenient							
Masters/Doctorate	1.925	1.657	-0.225	0.306	-0.144	0.299	456
Bachelors	1.942	2.167	0.137	0.335	-0.028	0.311	206
**By Experience**							
Harsh							
Above Median	1.492	1.542	0.196	0.235	0.247	0.172	360
Below Median	1.253	1.629	0.330***	0.079	0.334***	0.104	317
Lenient							
Above Median	2.122	1.877	-0.264	0.397	-0.198	0.405	319
Below Median	1.759	1.751	0.011	0.208	-0.017	0.216	343
**By Teachers' Locale**							
Harsh							
Non-Urban	1.294	1.600	0.223	0.232	0.251	0.228	224
Urban	1.432	1.482	0.241**	0.116	0.298**	0.122	431
Lenient							
Non-Urban	2.025	1.991	0.116	0.385	0.245	0.389	233
Urban	1.899	1.718	-0.248	0.283	-0.267	0.289	408

Notes: This table presents results from sub sample regressions by teachers' characteristics. The outcome is the grading fairness gap. All specifications include a comprehensive set of control variables, such as gender, age, level of education, teaching experience, years since graduation, an indicator for whether the participant studied abroad, baseline grading leniency, and indicators to reflect any missing values. We also control for teachers' field of specialization and include county fixed effects. Standard errors clustered at county level are reported in parentheses. *, ** and *** indicate statistical significance at the 10%, 5%, and 1% level, respectively. Median age is 51. Figure S5 plots the age distribution. Median experience is 21 years. Figure S6 plots the experience distribution.

Table S19: Differential Results by Teachers' Field of Specialization, Studied Abroad Status, Baseline Technological Literacy, and Leniency

	(1)	(2)	(3)	(4)	(5)	(6)	(7)
	Means		Without Controls		With Controls		
Scenario	Human	Algorithm	$\hat{\beta}$	SE	$\hat{\beta}$	SE	N
**Humanities Vs. STEM**							
Harsh							
Humanities	1.922	2.292	0.528***	0.172	0.533***	0.149	371
STEM	0.627	0.595	-0.080	0.106	0.020	0.124	151
Primary/Other	0.847	0.829	-0.037	0.091	0.011	0.094	155
Lenient							
Humanities	1.977	2.006	0.066	0.296	0.017	0.335	343
STEM	1.080	0.798	-0.231	0.247	-0.223	0.253	172
Primary/Other	2.886	2.494	-0.430	0.467	-0.281	0.340	147
**By Studied Abroad**							
Harsh							
Studied Abroad	1.694	1.634	0.030	0.274	-0.098	0.224	143
Didn't Study Abroad	1.276	1.625	0.375***	0.133	0.484***	0.123	461
Lenient							
Studied Abroad	2.377	2.077	-0.357	0.416	-0.521	0.476	126
Didn't Study Abroad	1.771	1.592	-0.136	0.241	-0.129	0.218	436
**By Tech Literacy**							
Harsh							
Above Median	0.985	1.483	0.437**	0.198	0.486**	0.189	403
Below Median	1.992	1.715	-0.011	0.156	0.013	0.150	276
Lenient							
Above Median	1.797	1.874	0.035	0.361	-0.020	0.349	380
Below Median	2.110	1.738	-0.316	0.256	-0.226	0.260	285
**By Leniency**							
Harsh							
Above Median	1.262	1.529	0.298***	0.111	0.310**	0.120	376
Below Median	1.486	1.625	0.249	0.217	0.337	0.221	292
Lenient							
Above Median	2.222	1.878	-0.328	0.364	-0.318	0.356	386
Below Median	1.508	1.703	0.160	0.160	0.157	0.196	268

Notes: This table presents results from sub sample regressions by teachers' characteristics. The outcome is the grading fairness gap. All specifications include a comprehensive set of control variables, such as gender, age, level of education, teaching experience, years since graduation, an indicator for whether the participant studied abroad, baseline grading leniency, and indicators to reflect any missing values. We also control for teachers' field of specialization and include county fixed effects. Standard errors clustered at county level are reported in parentheses. *, ** and *** indicate statistical significance at the 10%, 5%, and 1% level, respectively. *Baseline Tech Literacy* is measured on a scale from -5 (Completely Disagree) to 5 (Completely Agree), based on teachers' agreement with the statement *"I possess the necessary knowledge and appropriate tools to meet the modern technological demands of my role as an educator."* Median *Baseline Technological Literacy* score is 4. Median leniency score is 7. Figure S4 plots the distribution of the *Baseline Technological Literacy* measure.

Table S20: Differential Results by Teachers' Characteristics—Alternative Specifications Using Interaction Terms

	Grading Fairness Gap			
	(1)	(2)	(3)	(4)
	Harsh		Lenient	
**By Gender**				
Algorithm × Female	-0.137	-0.133	-0.254	-0.123
	(0.189)	(0.178)	(0.382)	(0.411)
Algorithm	0.357*	0.394**	0.050	-0.023
	(0.203)	(0.193)	(0.449)	(0.471)
Female	-0.049	0.027	-0.149	-0.227
	(0.212)	(0.241)	(0.234)	(0.244)
N	677	677	662	662
**By Age**				
Algorithm × Age > Median	-0.165	-0.212	-0.267	-0.278
	(0.256)	(0.235)	(0.246)	(0.248)
Algorithm	0.383***	0.463***	-0.084	-0.081
	(0.092)	(0.110)	(0.322)	(0.260)
Age > Median	0.112	-1.318	0.249	-3.352***
	(0.228)	(1.054)	(0.200)	(0.713)
N	606	606	563	563
**By Educational Attainment**				
Algorithm × Masters/Doctorate	0.278	0.463**	-0.386	-0.115
	(0.178)	(0.230)	(0.376)	(0.328)
Algorithm	0.077	-0.022	0.135	-0.028
	(0.174)	(0.222)	(0.349)	(0.327)
Masters/Doctorate	-0.447***	-1.826**	0.044	-0.002
	(0.149)	(0.805)	(0.204)	(0.396)
N	677	677	662	662
Controls	No	Yes	No	Yes

Notes: This table reports regression estimates that interact the treatment indicator with each teacher characteristic, allowing the treatment effect to vary across subgroups. The outcome is the grading fairness gap. All specifications include a comprehensive set of control variables, such as gender, age, level of education, teaching experience, years since graduation, an indicator for whether the participant studied abroad, baseline grading leniency, and indicators to reflect any missing values. We also control for teachers' field of specialization and include county fixed effects. Standard errors clustered at county level are reported in parentheses. *, ** and *** indicate statistical significance at the 10%, 5%, and 1% level, respectively.

Table S20: Differential Results by Teachers' Characteristics—Alternative Specifications Using Interaction Terms [Continued]

	(1)	(2)	(3)	(4)
	**Harsh**		**Lenient**	
**By Experience**				
Algorithm × Experience > Median	-0.128	-0.099	-0.244	-0.201
	(0.279)	(0.214)	(0.313)	(0.378)
Algorithm	0.324***	0.345***	-0.022	0.001
	(0.081)	(0.105)	(0.229)	(0.234)
Experience > Median	-0.043	-0.323**	0.246	0.174
	(0.201)	(0.139)	(0.222)	(0.252)
N	677	677	662	662
**By Teachers' Locale**				
Algorithm × Urban	0.012	0.047	-0.392	-0.512
	(0.274)	(0.285)	(0.489)	(0.549)
Algorithm	0.222	0.251	0.118	0.245
	(0.241)	(0.239)	(0.404)	(0.409)
Urban	-0.981	-1.423	0.567	0.673
	(0.983)	(0.943)	(0.561)	(0.671)
N	655	655	641	641
**Humanities Vs. STEM**				
Algorithm × Humanities	0.635***	0.489**	0.241	0.083
	(0.200)	(0.214)	(0.381)	(0.354)
Algorithm	-0.083	0.051	-0.153	-0.100
	(0.114)	(0.157)	(0.255)	(0.285)
Humanities	-0.119	0.063	1.352***	1.380***
	(0.151)	(0.151)	(0.255)	(0.433)
N	522	522	515	515
Controls	No	Yes	No	Yes

Notes: This table reports regression estimates that interact the treatment indicator with each teacher characteristic, allowing the treatment effect to vary across subgroups. The outcome is the grading fairness gap. All specifications include a comprehensive set of control variables, such as gender, age, level of education, teaching experience, years since graduation, an indicator for whether the participant studied abroad, baseline grading leniency, and indicators to reflect any missing values. We also control for teachers' field of specialization and include county fixed effects. Standard errors clustered at county level are reported in parentheses. *, ** and *** indicate statistical significance at the 10%, 5%, and 1% level, respectively.

Table S20: Differential Results by Teachers' Characteristics—Alternative Specifications Using Interaction Terms [Continued]

	Grading Fairness Gap			
	(1)	(2)	(3)	(4)
	Harsh		Lenient	
**By Studied Abroad**				
Algorithm × Studied Abroad	-0.341	-0.581**	-0.317	-0.392
	(0.276)	(0.244)	(0.371)	(0.445)
Algorithm	0.372**	0.484***	-0.134	-0.129
	(0.140)	(0.129)	(0.254)	(0.232)
Studied Abroad	0.335**	0.434**	0.586	0.698*
	(0.153)	(0.189)	(0.368)	(0.413)
N	604	604	562	562
**By Baseline Tech Literacy**				
Algorithm × Tech Lit > Median	0.442	0.473	0.399	0.206
	(0.289)	(0.289)	(0.342)	(0.338)
Algorithm	-0.009	0.013	-0.362	-0.226
	(0.162)	(0.157)	(0.283)	(0.273)
Tech Lit > Median	-0.410**	-0.378**	-0.272	-0.182
	(0.188)	(0.166)	(0.189)	(0.187)
N	677	677	662	662
**By Leniency**				
Algorithm × Leniency > Median	0.045	-0.027	-0.515	-0.474
	(0.249)	(0.288)	(0.342)	(0.318)
Algorithm	0.249	0.337	0.159	0.157
	(0.226)	(0.231)	(0.169)	(0.207)
Leniency > Median	-0.111	0.251	0.678***	1.170*
	(0.167)	(0.454)	(0.166)	(0.691)
N	668	668	654	654
Controls	No	Yes	No	Yes

Notes: This table reports regression estimates that interact the treatment indicator with each teacher characteristic, allowing the treatment effect to vary across subgroups. The outcome is the grading fairness gap. All specifications include a comprehensive set of control variables, such as gender, age, level of education, teaching experience, years since graduation, an indicator for whether the participant studied abroad, baseline grading leniency, and indicators to reflect any missing values. We also control for teachers' field of specialization and include county fixed effects. Standard errors clustered at county level are reported in parentheses. *, ** and *** indicate statistical significance at the 10%, 5%, and 1% level, respectively.

Table S21: Differential Mechanisms Results by Teachers' Characteristics—Using Interaction Terms

	Ability	Comprehension	Fairness	Intent	Responsibility	Ability	Comprehension	Fairness	Intent	Responsibility
	(1)	(2)	(3)	(4)	(5)	(6)	(7)	(8)	(9)	(10)
	Harsh					Lenient				
**By Age**										
Algorithm × Age > Median	1.029**	-0.172	0.047	0.151	0.361	0.349	-0.494	-0.782*	-0.567	-0.279
	(0.416)	(0.553)	(0.633)	(0.543)	(0.541)	(0.453)	(0.553)	(0.406)	(0.401)	(0.494)
Algorithm	-2.346***	0.466	-0.513	-1.780***	-1.924***	-2.386***	-0.577	-0.539	-1.741***	-1.732***
	(0.370)	(0.519)	(0.672)	(0.441)	(0.536)	(0.319)	(0.656)	(0.496)	(0.629)	(0.608)
Age > Median	4.681	1.483	4.252	5.121	3.169	-0.538	1.888	0.079	1.784	0.004
	(3.546)	(3.898)	(5.185)	(4.189)	(4.544)	(2.713)	(1.736)	(2.192)	(1.105)	(1.693)
N	606	606	606	606	606	563	563	563	563	563
**By Educational Attainment**										
Algorithm × Masters/Doctorate	-0.157	0.852**	0.645	1.243***	-0.059	-0.823	0.227	0.375	-0.277	-0.011
	(0.464)	(0.346)	(0.439)	(0.426)	(0.526)	(0.519)	(0.805)	(0.557)	(0.390)	(0.791)
Algorithm	-1.670***	-0.211	-0.942**	-2.548***	-1.673***	-1.684***	-0.978	-1.141*	-1.720***	-1.834*
	(0.408)	(0.413)	(0.403)	(0.460)	(0.424)	(0.514)	(0.814)	(0.636)	(0.578)	(0.932)
Masters/Doctorate	-2.847***	1.902	1.294	2.065	1.825	0.843*	0.925**	-0.152	0.102	0.019
	(0.725)	(1.928)	(1.667)	(2.247)	(2.747)	(0.496)	(0.413)	(0.588)	(0.599)	(0.596)
N	615	615	615	615	615	582	582	582	582	582
**By Baseline Tech Literacy**										
Algorithm × Tech Lit > Median	-0.136	0.490	0.333	0.388	-0.028	-0.319	0.263	0.442	-0.286	0.627
	(0.584)	(0.458)	(0.495)	(0.441)	(0.600)	(0.399)	(0.855)	(0.396)	(0.441)	(0.405)
Algorithm	-1.693***	0.080	-0.696***	-1.928***	-1.695***	-2.094***	-0.954	-1.149**	-1.751***	-2.185***
	(0.313)	(0.400)	(0.216)	(0.229)	(0.314)	(0.408)	(0.872)	(0.563)	(0.611)	(0.632)
Tech Lit > Median	0.248	-0.251	-0.001	-0.532*	0.149	-0.178	0.046	-0.753**	0.241	-0.482
	(0.435)	(0.358)	(0.304)	(0.302)	(0.411)	(0.368)	(0.617)	(0.336)	(0.311)	(0.407)
N	615	615	615	615	615	582	582	582	582	582
**Humanities Vs. STEM**										
Algorithm × Humanities	-2.191***	-1.197**	-1.481**	-2.298***	-1.330**	0.727	1.263	0.103	-0.334	0.003
	(0.617)	(0.557)	(0.616)	(0.544)	(0.525)	(0.892)	(1.339)	(0.820)	(0.496)	(0.922)
Algorithm	-0.295	0.799	0.445	-0.219	-0.979*	-2.922***	-1.804***	-1.212*	-1.840***	-2.025***
	(0.396)	(0.520)	(0.534)	(0.439)	(0.500)	(0.538)	(0.673)	(0.661)	(0.507)	(0.549)
Humanities	1.486***	2.163***	2.586***	2.451***	2.277***	0.778	1.166	1.638**	1.452**	1.383**
	(0.475)	(0.470)	(0.482)	(0.499)	(0.480)	(0.701)	(0.905)	(0.615)	(0.597)	(0.614)
N	477	477	477	477	477	448	448	448	448	448
Controls	Yes	Yes	Yes	Yes	Yes	Yes	Yes	Yes	Yes	Yes

Notes: This table reports regression estimates on teacher perceptions using interactions between the treatment indicator am each teacher characteristic, allowing the treatment effect to vary across subgroups. All specifications include a comprehensive set of control variables, such as gender, age, level of education, teaching experience, years since graduation, an indicator for whether the participant studied abroad, baseline grading leniency, and indicators to reflect any missing values. We also control for teachers' field of specialization and include county fixed effects. Standard errors clustered at county level are reported in parentheses. *, ** and *** indicate statistical significance at the 10%, 5%, and 1% level, respectively.

Table S22: Mediation Analysis, Humanities Teachers

	Mediators									
	Ability		Comprehension		Fairness		Intent		Responsibility	
Scenario	$\hat{\beta}$	P value	$\hat{\beta}$	P value	$\hat{\beta}$	P value	$\hat{\beta}$	P value	$\hat{\beta}$	P value
**Harsh**										
Indirect	0.500	0.001	0.024	0.457	0.128	0.014	0.179	0.054	0.446	0.000
	(0.216 to 0.784)		(-0.039 to 0.087)		(0.026 to 0.230)		(-0.003 to 0.361)		(0.233 to 0.659)	
Direct	0.180	0.334	0.655	0.000	0.550	0.003	0.501	0.022	0.238	0.195
	(-0.185 to 0.544)		(0.321 to 0.988)		(0.191 to 0.909)		(0.071 to 0.931)		(-0.122 to 0.598)	
Mediator Mean	0.506		0.573		-0.365		-0.249		-0.749	
Mediator SD	3.429		3.506		3.344		3.231		3.410	
**Lenient**										
Indirect	-0.320	0.005	-0.063	0.544	-0.225	0.127	-0.505	0.001	-0.349	0.051
	(-0.544 to -0.096)		(-0.268 to 0.141)		(-0.514 to 0.064)		(-0.803 to -0.207)		(-0.699 to 0.001)	
Direct	0.245	0.611	-0.015	0.961	0.144	0.672	0.426	0.386	0.268	0.440
	(-0.699 to 1.189)		(-0.630 to 0.599)		(-0.523 to 0.812)		(-0.537 to 1.389)		(-0.412 to 0.948)	
Mediator Mean	0.680		0.743		-0.657		-0.180		-0.760	
Mediator SD	3.449		3.453		3.359		3.276		3.571	

Parameter $\hat{\beta}$ is the estimated parameter of interest from mediation analysis. A positive $\hat{\beta}$ indicates that the algorithmic recommendation increased the gap relative to the human recommendation; a negative $\hat{\beta}$ indicates that it reduced the gap. Mediator questions were presented after outcome questions in our survey instrument. *Grading fairness gap* is the absolute difference between a teacher's grade and an objective or benchmark grade, and is used to measure the extent of deviation from fair grading. All specifications include a comprehensive set of control variables, such as gender, age, level of education, teaching experience, years since graduation, an indicator for whether the participant studied abroad, baseline grading leniency, and indicators to reflect any missing values. We also control for teachers' field of specialization and include county fixed effects. Standard errors are clustered at county level. Confidence intervals at the 95% level are reported in parentheses.

Table S23: Teacher Attitudes and Use of Artificial Intelligence

	%		N
Do you use generative AI tools like ChatGPT to prepare your lessons?			
More than once a week	48.22		569
About once a week	7.88		93
One to three times a month	5.25		62
Less than once a month	12.71		150
Never	25.93		306
Do you encourage your students to use generative AI tools like ChatGPT?			
Unreservedly	17.53		207
Yes, but with reservations	23.96		283
I neither encourage nor discourage it	40.98		484
No, but there are exceptions	16.43		194
Absolutely not	1.10		13
Do you encourage your fellow teachers to use generative AI tools like ChatGPT?			
Unreservedly	6.35		75
Yes, but with reservations	9.56		113
I neither encourage nor discourage it	27.83		329
No, but there are exceptions	48.48		573
Absolutely not	7.78		92
Rated from -5 (Definitely No/Unethical) to 5 (Definitely Yes/Ethical)	Mean	SD	N
Do you believe that AI systems can grade fairly?	0.03	2.81	1,113
Would you use AI tools for grading?	-1.03	3.18	1,146
How ethical do you think it is for student's work to be graded by AI systems?	-1.33	3.03	1,119

Notes: This table presents summary statistics on teachers' responses regarding their use of and attitudes toward artificial intelligence in teaching. Figure S9 plots the distributions of teacher responses on three dimensions of AI grading: beliefs about its fairness (Panel A), willingness to use AI tools for grading (Panel B), and perceived ethics on the use of AI to assess students (Panel C).

Table S24: Sample Representativeness

		Greek Census	
	Survey Sample	K-12 Teachers	All Employed
Women (%)	69	72	38
Mean Age (Yrs)	49	40	41
Mean Experience (Yrs)	20	18	19
Education (%)			
Bachelors/Masters	92	98	99.4
Doctorate	8	2	0.6

Notes: The table compares key demographic and educational characteristics of the survey sample with K–12 teachers and the overall employed population in Greece. Education refers to highest educational attainment.The Census data are sourced from the Hellenic Statistical Authority (ELSTAT).

S1 Supplementary Survey

To address concerns about potential platform-specific idiosyncrasies and noise in online survey data, we conducted a supplementary replication of the experiment using the same survey instrument, platform (Qualtrics) and recruitment procedure. This additional wave collected responses from 94 teachers in February 2026. Appendix Table S25 reports balance statistics, showing that treatment assignment remains broadly comparable across observable characteristics. Next, Appendix Table S26 presents regression estimates using a specification, similar to the one presented in Appendix Table S7 for the main sample, that includes the key interaction between the algorithm treatment and the harsh scenario. Consistent with the main results, the harsh scenario generates significantly larger grading deviations, and the interaction term $Algorithm \times Harsh\ Scenario$ is positive, statistically significant, and its magnitude is larger than in the baseline estimates ($\hat{\beta} = 1.575$, SE=0.748 versus $\hat{\beta} = 0.417$, SE=0.167 in the main sample). While these estimates should be interpreted as exploratory due to the smaller sample size, they provide additional reassurance that the core pattern observed in the main analysis is not driven by a particular sample collection.

Table S25: Supplementary Survey - Respondent Characteristics and Balance Tests

	(1)	(2)	(3)	(4)	(5)	(6)	(7)	(8)
		Human			Algorithm			
	Mean	SD	*N*	Mean	SD	*N*	Diff.	*P* value
Age (Yrs)	47.7	11.0	39	48.0	10.0	46	0.377	0.870
Experience (Yrs)	18.7	11.3	42	16.8	11.9	52	-1.840	0.446
Years Since Graduation (Yrs)	27.1	11.7	42	23.2	10.8	52	-3.908	0.100
Baseline Leniency (0-10)	6.8	2.0	42	7.1	1.8	51	0.312	0.434
	%		*N*	%		*N*	Diff.	*P* value
Gender								
Female	71.4		42	67.3		52	-4.121	0.670
Male	28.6		42	32.7		52	4.121	0.670
Education								
Bachelors	35.7		42	32.7		52	-3.022	0.762
Masters	57.1		42	59.6		52	2.473	0.812
Doctorate	4.8		42	5.8		52	1.007	0.829
Studied Abroad	38.5		39	28.3		46	-10.201	0.328
Field								
Humanities	54.8		42	50.0		52	-4.762	0.650
STEM	26.2		42	19.2		52	-6.960	0.432
Primary/Other	19.0		42	30.8		52	11.722	0.191
**Baseline Technological Literacy**								
"I possess the necessary knowledge and appropriate tools to meet the modern technological demands of my role as an educator."								
	Mean	SD	*N*	Mean	SD	*N*	Diff.	*P* value
Score	3.4	1.8	42	2.9	2.1	52	-0.492	0.222

Notes: The table reports summary statistics for 94 teachers in the Supplementary Survey we disseminated during February 2026 across the treatment conditions *Human* and *Algorithm*. *Human* refers to the treatment condition in which a participant was exposed to a human recommender. *Algorithm* refers to a treatment condition in which a participant was exposed to an algorithmic recommender. Field denotes the teacher's area of specialization and is categorized as follows: STEM, which encompasses subjects such as mathematics, physics, chemistry, science, and computer science; Humanities, which includes disciplines such as theology, art, sociology, and economics; and Primary/Other, which refers to educators involved in Greek language instruction, primary education, or teaching in technical institutions. *P* values stemming from a two-sample mean comparison t test are reported. Leniency is assessed on a scale from 0 to 10 based on the grades assigned by participants in the baseline exercises. Self-reported *Baseline Technological Literacy* is measured on a scale from -5 (Completely Disagree) to 5 (Completely Agree). Sample sizes vary slightly across rows due to item non-response.

Table S26: Supplementary Survey - Algorithm × Harsh Scenario Interaction Effects on Grading Fairness Gap

	Grading Fairness Gap
	(1)
Algorithm × Harsh Scenario	1.575**
	(0.748)
Algorithm	-0.593
	(0.520)
Harsh Scenario	-4.469***
	(0.520)
N	94
Controls	Yes

Notes: This table reports estimates from a multiple regression specification. *Grading fairness gap* is the absolute difference between a teacher's grade and an objective or benchmark grade, and is used to measure the extent of deviation from fair grading. All specifications include teacher field of specialization fixed effects and a comprehensive set of control variables, such as gender, age, level of education, teaching experience, years since graduation, an indicator for whether the participant studied abroad, baseline grading leniency, and indicators to reflect any missing values. Standard errors clustered at county level are reported in parentheses. * $p < 0.1$; ** $p < 0.05$; *** $p < 0.01$.

S2 Questionnaire

S2.1 Baseline Teacher Characteristics

Q1. What is your gender?

- Man
- Woman
- Other
- Prefer not to answer

Q2. In what year did you complete your first cycle of studies?

(Dropdown selection: e.g., 2000)

Q3. In what year did you begin teaching as a full-time employee?

(Dropdown selection: e.g., 2000)

Q4. What is the highest academic degree you hold?

- High school diploma or still studying in college
- Bachelor's degree from a Higher Educational Institution
- Master's degree from a Higher Educational Institution
- Doctorate from a Higher Educational Institution

Q5. Have you studied abroad?

- Yes
- No

Q6. What was your specialty upon first appointment?

(Dropdown selection: e.g., PE01)

Q7. To what extent do you agree with the following statement?

I possess the necessary knowledge and appropriate tools to meet the modern technological demands of my role as an educator.

- **-5** means **Strongly Disagree**
- **+5** means **Strongly Agree**

(Response scale: -5 to +5 with slider)

S2.2 Baseline Leniency Measures

Mathematics and Physics Teachers

A student was asked to solve the following exercise.

Q8. What grade would you give out of 10? *(Response scale: 0 to 10 with slider)*

Question: *"In a right triangle, one leg is* $a = 8$ *cm, and the other leg is* $b = 6$ *cm. Calculate the hypotenuse c."*

Student's Answer:

$$c^2 = a^2 + b^2$$
$$c^2 = 8^2 + 6^2$$
$$c^2 = 64 + 36$$
$$c^2 = 100$$
$$c = 9$$

(The student applied the correct formula but made a mistake extracting the square root of 100; the correct answer is $c = 10$*.)*

Science Teachers

A student was asked to solve the following exercise.

Q8. What grade would you give out of 10? *(Response scale: 0 to 10 with slider)*

Question: *"Balance the following chemical equation:*

$$H_2 + O_2 \rightarrow H_2O$$

Explain your process."

Student's Answer:

$$H_2 + O_2 \rightarrow 2H_2O$$

(The equation is incorrectly balanced. The correct balanced equation is:

$2H_2 + O_2 \rightarrow 2H_2O$*. The student did not provide an explanation of their process either.)*

Non-STEM Teachers

A student was asked to respond to the following exercise.

Q8. What grade would you give out of 10? *(Response scale: 0 to 10 with slider)*

Question: *"Identify the syntactic components of the sentence:*

≪Technology facilitates the life of humans in many aspects of daily life.≫"

Student's Answer:

- Technology: Subject
- facilitates: Object (Incorrect — this is actually the verb/predicate)
- the life: Object (Correct)
- of humans: Genitive modifier (Correct)
- in many aspects: Adverbial modifier of place (Correct)

S2.3 Randomization Blocks

S2.3.1 Mathematics and Physics Teachers, Harsh Scenario

The following exercise was graded by an [**algorithmic grading system or colleague**] with a score of 5 on a scale from 0 to 10.

Question:

"In an isosceles triangle $\triangle ABC$, the angles have the following characteristics:

- $\angle A = 40°$
- $\angle B = \angle C$

Identify:

1. The base.
2. The equal sides.
3. The equal angles.
4. The vertex angle.
5. The sum of the angles of the triangle."

Answer:

1. **Base:** BC. (Correct)
2. **Equal sides:** $AB = AC$. (Correct)
3. **Equal angles:** $\angle B = \angle C$. (Correct)
4. **Vertex angle:** $\angle A$. (Correct)
5. **Sum of angles:** $100°$. (Incorrect)

S2.3.2 Mathematics and Physics Teachers, Lenient Scenario

The following exercise was graded by an [**algorithmic grading system or colleague**] with a score of 5 on a scale from 0 to 10.

Question:

"In an isosceles triangle $\triangle ABC$, the angles have the following characteristics:

- $\angle A = 40°$
- $\angle B = \angle C$

Identify:

1. The base.
2. The equal sides.
3. The equal angles.
4. The vertex angle.
5. The sum of the angles of the triangle."

Answer:

1. **Base:** BC. (Correct)
2. **Equal sides:** $AB = BC$. (Incorrect)
3. **Equal angles:** $\angle A = \angle B$. (Incorrect)
4. **Vertex angle:** $\angle B$. (Incorrect)
5. **Sum of angles:** $100°$. (Incorrect)

S2.3.3 Greek Philologists, Harsh Scenario

The following exercise was graded by an [**algorithmic grading system or colleague**] with a score of 5 on a scale from 0 to 10.

Question:

"Syntactically analyze the sentence:

Τὸ γὰρ εὖ ζῆν τῷ εὖ πράττειν ταὐτόν ἐστιν.

Answer:

- **Τὸ:** Article. (Correct)
- **γὰρ:** Conjunction. (Correct)
- **εὖ:** Adverb. (Correct)
- **ζῆν:** Infinitive. (Correct)
- **ταὐτόν:** Subject. (Incorrect)

S2.3.4 Greek Philologists, Lenient Scenario

The following exercise was graded by an [**algorithmic grading system or colleague**] with a score of 5 on a scale from 0 to 10.

Question:

"Syntactically analyze the sentence:

Τὸ γὰρ εὖ ζῆν τῷ εὖ πράττειν ταὐτόν ἐστιν.

Answer:

- **Τὸ:** Article. (Correct)
- **γὰρ:** Adverb. (Incorrect)
- **εὖ:** Adjective. (Incorrect)
- **ζῆν:** Verb. (Incorrect)
- **ταὐτόν:** Subject. (Incorrect)

S2.3.5 Other Non-STEM, Harsh Scenario

The following exercise was graded by an [**algorithmic grading system or colleague**] with a score of 5 on a scale from 0 to 10.

Question:

"Read the following passage and answer the questions:

Text:

"Friendship is a precious treasure in life. Through it, people learn the meaning of mutual support and trust, while they share moments of joy and sorrow."

1. What is the theme of the passage?
2. Identify the type of the text (description, narration, argumentation).
3. Find one word from the text that expresses emotion.
4. Write a title for the text.
5. What is the significance of friendship as described in the text?

Answer:

1. **Theme of the passage:** Friendship. (Correct)
2. **Text type:** Argumentation. (Correct)
3. **Word expressing emotion:** Joy. (Correct)
4. **Title for the text:** "The value of friendship." (Correct)
5. **Significance of friendship:** Friendship is important because it brings trust, mutual support, joy, and sorrow. (Incorrect: The answer omits the connection with "life as a treasure.")

S2.3.6 Other Non-STEM, Lenient Scenario

The following exercise was graded by an [**algorithmic grading system or colleague**] with a score of 5 on a scale from 0 to 10.

Question:

"Read the following text and answer the questions:

Text:

"Friendship is a precious treasure in life. Through it, people learn the meaning of mutual support and trust, while sharing moments of joy and sorrow."

1. What is the theme of the text?
2. Identify the type of the text (description, narration, argumentation).
3. Find one word in the text that expresses emotion.
4. Write a title for the text.
5. What is the meaning of friendship, as described in the text?

Answer:

1. **Theme of the text:** Friendship. (Correct)
2. **Text type:** Narration. (Incorrect: The correct answer is argumentation, as the text develops an argument.)
3. **Word expressing emotion:** Treasure. (Incorrect: The correct answer would be "joy" or "sorrow.")
4. **Title for the text:** "The value of trust." (Incorrect: The title is misleading, as it doesn't focus on friendship. A correct title would be "The value of friendship.")
5. **Meaning of friendship:** Friendship is essential because it brings trust. (Incorrect: The answer is incomplete and does not include joy, sorrow, and mutual support as mentioned in the text.)

S2.3.7 Science, Harsh Scenario

The following exercise was graded by an [**algorithmic grading system or colleague**] with a score of 5 on a scale from 0 to 10.

Question:

"Read the following statement and answer the questions:

Statement:

"The lungs are the primary organs of the respiratory system. Through them, oxygen and carbon dioxide are exchanged between the air and the blood."

1. What is the role of the lungs?
2. In which system are the lungs found?
3. What gases are exchanged through the lungs?
4. What is the main muscle that assists with breathing?
5. What is the primary function of blood in gas exchange?

Answer:

1. **Role of the lungs:** To carry out the exchange of oxygen and carbon dioxide with the blood. (Correct)
2. **System:** Respiratory. (Correct)
3. **Gases:** Oxygen and carbon dioxide. (Correct)
4. **Muscle:** Diaphragm. (Correct)
5. **Function of the blood:** It only transports carbon dioxide from the tissues to the lungs. (Incorrect: The blood transports both carbon dioxide from the tissues to the lungs and oxygen from the lungs to the tissues.)

S2.3.8 Science, Lenient Scenario

The following exercise was graded by an [**algorithmic grading system or colleague**] with a score of 5 on a scale from 0 to 10.

Question:

"Read the following statement and answer the questions:

Statement:

"The lungs are the primary organs of the respiratory system. Through them, oxygen and carbon dioxide are exchanged between the air and the blood."

1. What is the role of the lungs?
2. In which system are the lungs found?
3. What gases are exchanged through the lungs?
4. What is the main muscle that assists with breathing?
5. What is the primary function of blood in gas exchange?

Answer:

1. **Role of the lungs:** To produce oxygen. (Incorrect: The role of the lungs is to exchange oxygen and carbon dioxide with the blood.)
2. **System:** Circulatory. (Incorrect: The lungs belong to the respiratory system.)
3. **Gases:** Only oxygen. (Incorrect: The gases exchanged are oxygen and carbon dioxide.)
4. **Muscle:** Lungs. (Incorrect: The main muscle that assists with breathing is the diaphragm.)
5. **Function of the blood:** To transport oxygen to the tissues. (Correct: The blood transports oxygen to the tissues, including the muscles.)

S2.4 Primary Outcome: Teacher Grade

Q9. What score would you give out of 10? *(Response scale: 0 to 10 with slider)*

S2.5 Secondary Outcomes: Mediators

How much do you agree with the following statements?

Q10. I believe that the [**algorithmic grading system or colleague**] who graded the above assignment has the student's best interest in mind.

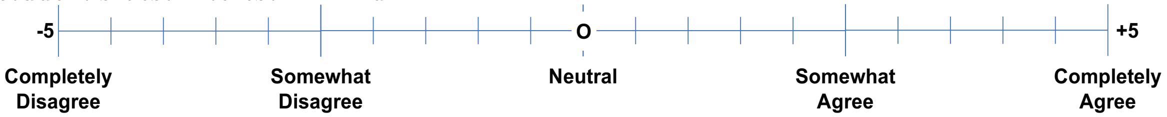

Q11. I believe that the [**algorithmic grading system or colleague**] who graded the above assignment is fair.

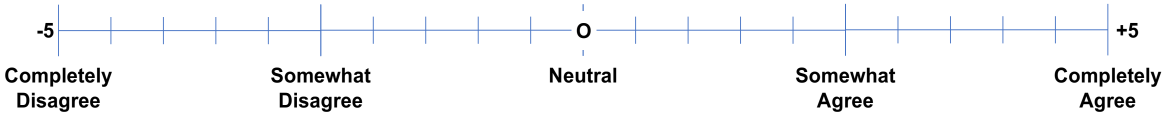

Q12. I believe that the [**algorithmic grading system or colleague**] who graded the above assignment has sufficient knowledge of the subject.

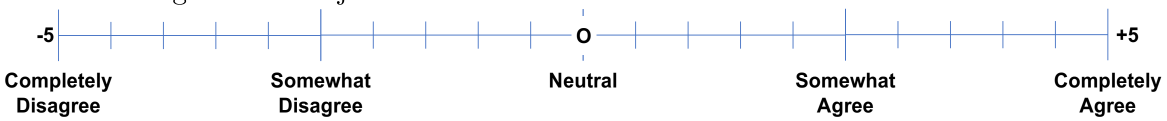

Q13. I understand why the [**algorithmic grading system or colleague**] who graded the above assignment gave that grade.

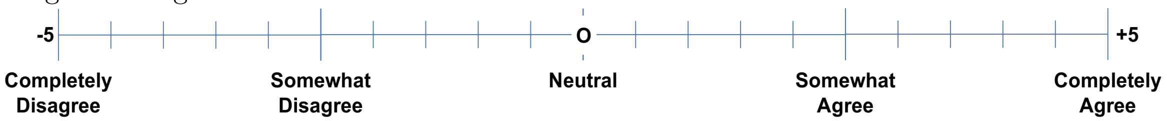

Q14. I believe that the [**algorithmic grading system or colleague**] who graded the above assignment is a responsible grader.

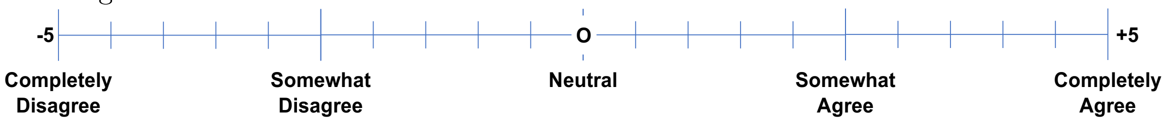

S2.6 Artificial Intelligence Use in Teaching Practice

Q15. Do you use generative artificial intelligence tools like ChatGPT to prepare your lessons?

- More than once a week
- About once a week
- One to three times a month
- Less than once a month
- Never

Q16. Do you encourage your students to use generative artificial intelligence tools like ChatGPT?

- Unreservedly
- Yes, but with reservations
- I neither encourage nor discourage it
- No, but there are exceptions
- Absolutely not

Q17. Would you encourage your fellow teachers to use generative artificial intelligence tools like ChatGPT?

- Unreservedly
- Yes, but with reservations
- I would neither encourage nor discourage it
- No, but there are exceptions
- Absolutely not

Q18. Would you use AI tools for grading?

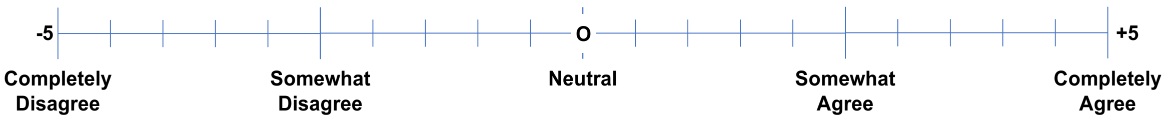

Q19. How ethical do you think it is for students' work to be graded by AI systems?

Q20. Do you believe that AI systems can grade fairly?

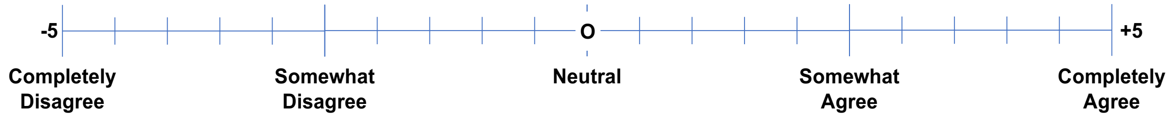

Q21. Is there anything you would like to add or explain?
